# Identification and validation of depression-associated genetic variants in the UK Biobank cohort with transcriptome and DNA methylation analyses in independent cohorts

**DOI:** 10.1016/j.heliyon.2025.e41865

**Published:** 2025-01-10

**Authors:** Muataz S. Lafta, Aleksandr V. Sokolov, Gull Rukh, Helgi B. Schiöth

**Affiliations:** Department of Surgical Sciences, Functional Pharmacology and Neuroscience, Uppsala University, Uppsala, Sweden

**Keywords:** UK biobank, Depression, Independent genetic variants, Genetic risk score, Transcriptome, Methylation

## Abstract

Depression is one of the most common psychiatric conditions resulting from a complex interaction of genetic, epigenetic and environmental factors. The present study aimed to identify independent genetic variants in the protein-coding genes that associate with depression and to analyze their transcriptomic and methylation profile. Data from the GWAS Catalogue was used to identify independent genetic variants for depression. The identified genetic variants were validated in the UK Biobank cohort and used to calculate a genetic risk score for depression. Data was also used from publicly available cohorts to conduct transcriptome and methylation analyses. Eight SNPs corresponding to six protein-coding genes (*TNXB, NCAM1, LTBP3, BTN3A2, DAG1, FHIT*) were identified that were highly associated with depression. These validated genetic variants for depression were used to calculate a genetic risk score that showed a significant association with depression (p < 0.05) but not with co-morbid traits. The transcriptome and methylation analyses suggested nominal significance for some gene probes (*TNXB*- and *NCAM1*) with depressed phenotype. The present study identified six protein-coding genes associated with depression and primarily involved in inflammation (*TNXB*), neuroplasticity (*NCAM1 and LTBP3),* immune response (*BTN3A2),* cell survival (*DAG1) and* circadian clock modification (*FHIT)*. Our findings confirmed previous evidence for *TNXB*- and *NCAM1* in the pathophysiology of depression and suggested new potential candidate genes (*LTBP3, BTN3A2, DAG1* and FHIT*)* that warrant further investigation.

## Highlights

• Data was used from the GWAS Catalogue and validated in the UK Biobank cohort.
• Eight SNPs corresponding to six protein coding genes were highly associated with depression.
• The gene list includes *TNXB, NCAM1, LTBP3, BTN3A2, DAG1 and FHIT*.
• A genetic risk score specific for depression was created based on the identified genes.
• The transcriptome and methylation analyses suggested nominal significance for *TNXB*- and *NCAM1* with depressed phenotype.

## 1 Introduction

Depression is one of the most common psychiatric conditions in the general population with an increasing global prevalence. It is a leading cause of functional impairment with a heavy disease burden reducing the quality of life of patients [1]. The lifetime morbidity of depression ranges from 20 to 25 % in women and 7–12 % in men [2]. It also poses higher risk for development of many severe medical illnesses [3]. At its worst, depression can significantly increase the risk for suicide [4,5]. In addition, studies on the economic burden for society have revealed huge costs for depression, both directly due to the illness itself, and indirectly due to loss of productivity and unemployment [6]. Therefore, research on depression has gained much attention over the last few decades to help reduce the global burden of the disease. Yet after decades of intensive research, depression remains a complex disorder with poorly understood mechanisms and differential presentation, clinical course and response to treatment among patients.

Depression is a complex disorder resulting from both genetic and environmental factors [7]. Several well-known environmental risk factors include mental stress, exposure to adverse life events, and addictive drug abuse, among others [8]. Interestingly, the environmental component, such as the exposure to stressful life events, can be influenced by genetic factors [9]. While the environmental risk factors might be well-established, the genetic associations are still not fully explored which places big focus on genetics. It has been suggested that common single nucleotide polymorphisms (SNPs) contribute about 9 % to the variation in liability [10]. Twin and family studies comparing concordance rates for major depression between monozygotic and dizygotic twins suggest that there is a heritability of about 37 % [7]. The heritability is even higher for some other forms of depression with early-onset or recurrent episodes [11]. Studies of families have also clearly shown the complex polygenic architecture of depression with combined effect from multiple genetic risk variants, each with a small effect size [12]. Technological advances in more recent years made it possible to move beyond family-based heritability studies in search of specific genetic variants involved in depression.

Numerous large-scale genome-wide association studies (GWAS) have been carried out to identify genetic risk variants linked with depression [[13], [14], [15], [16]]. One of the most recent and largest GWAS meta-analyses of depression, which included 807,533 individuals, identified 102 SNPs in/near 269 genes that show association with the disorder [17]. Despite many promising findings, identifying the risk loci through GWAS has proven challenging due to the complexity of the depression [15], and the achievements have been less successful than in other complex disorders such as schizophrenia and bipolar disorder [18,19]. There is even another challenge regarding how the identified risk variants affect pathogenesis and modulate the risk of depression. But given the fact that many of the reported risk variants are located in non-coding region, it is possible that they influence gene expression through a complex connection with transcriptional and epigenetic regulation.

During the past years, GWAS have provided evidence of associations between DNA methylation and other chromatin marks with nearby SNPs [[20], [21], [22]]. The interaction between genetic and epigenetic mechanisms may change vulnerability to depression by shifts in methylation and microRNA (mRNA) levels with relevance for gene expression. DNA methylation regulates transcriptional activity of a gene by influencing the packing of the DNA and its availability for transcription factors. Higher levels of methylation are associated with transcriptional repression by inhibiting the binding of transcription factors or by changing mRNA expression [23]. DNA methylation plays a critical role in normal brain development and function such as synaptic formation and function [24,25] and is strongly involved in several cognitive processes, such as learning and memory [26]. Perturbations in these chromatin and gene regulatory mechanisms, especially during adolescence may contribute to the susceptibility of psychiatric disorders [27]. Moreover, evidence also indicates the influence of age [28], sex [29], genetic background [30], and environmental exposure, such as social stress [31,32], on DNA methylation. These findings highlight the importance of epigenetics to interindividual variations in human disease such as depression and other co-morbid psychiatric traits.

In the current study, we aimed to identify independent genetic variants in the protein-coding genes that associate with depression. We selected depression-related SNPs from the GWAS Catalog and validated them in UK Biobank cohort. Based on the results from the UK Biobank data, we used the depression-related SNPs to create a genetic risk score as a prediction tool to enhance personalized treatment for depression and other co-morbid traits. We then used publicly available cohorts and conducted transcriptome analyses for the genes linked to the identified SNPs to identify differentially expressed transcripts between depressed individuals and controls. Finally, we investigated the depression-related genes at the epigenetic level and sought to identify potential changes in methylation sites among those genes.

## 2 Methods

### 2.1 UK Biobank

The current study was conducted based on data obtained from the United Kingdom (UK) Biobank resource. The UK Biobank is a large population-based cohort including rich health‐related data on more than 500,000 individuals with a roughly even number of men and women from the UK, aged between 40 and 69 years at the time of recruitment. The cohort was built as a resource to enable studies on diseases of middle and old age and the recruitment of participants took place between 2006 and 2010 at multiple centers across England, Scotland and Wales. During the initial visit, baseline assessments were conducted including clinical measurements, biological samples and questionnaires and verbal interviews to gather sociodemographic, lifestyle-related and medical information [33]. All the participants provided informed written consent for their data to be used in future research with the possibility to withdraw at any time and the UK Biobank project has been approved by The UK Biobank Research Ethics Committee (REC), (REC reference 11/NW/0382). This study was conducted using the UK Biobank Resource under application number 30172 and for our use of UK Biobank data, an approval was obtained by Regional Ethics Committee of Uppsala, Sweden (2017/198). All methods were performed in accordance with the relevant guidelines and regulations.

### 2.2 Genotype data

We used the GWAS Catalog database to identify SNPs associated with depression in European cohorts. GWAS Catalog is a publicly available database of published human GWAS investigating associations between SNPs and a variety of phenotypes, including many psychiatric disorders [34]. We used a broad definition of depression including different types of depression and applied specific exclusion terms (Table S12) to include only depression-specific SNPs in European cohorts and exclude variants associated with other traits. We initially located 1039 associations reported in depression-related GWAS (European population), which were mapped to 712 unique genes. Mapping of SNPs to genes was based on SNP positions and provided by the GWAS Catalog database preprocessing pipeline, column “MAPPED GENE". Out of all SNPs in GWAS Catalog that were associated with depression, we selected only those SNPs whose genes were measurable by Olink Proteomics to enable proteomic studies on those genes in future research. Our strategy resulted in a total of 54 SNPs.

In the next step, we extracted the identified depression-specific SNPs from the UK biobank genotype data. In the UK Biobank, genotype data were available for 487,409 participants. Genome-wide genotyping was conducted using the Applied Biosystems UK BiLEVE Axiom Array by Affymetrix (now part of Thermo Fisher Scientific) and the Applied Biosystems UK Biobank Axiom Array [35]. The extracted SNPs underwent quality control and all the SNPs with minor allele frequency (MAF) ≤ 1 %, genotyping rate <0.988 and/or missing rate >10 % were excluded yielding a total of 47 SNPs. For those 47 SNPs, independent variants were selected by testing the linkage disequilibrium (LD) within each SNP pair using LD matrix via LDlink platform (SNPs with r2 < 0.8 were selected) [36], which led to the exclusion of 8 SNPs. Lastly, the SNPs that deviated from Hardy-Weinberg equilibrium at the significance level of 5.7 × 10^−7^ [37] were excluded, leaving 38 SNPs that were included in our analyses. A summary of all the steps to create the genotype data is shown in Fig. S1.

### 2.3 Phenotype data

#### 2.3.1 Cases and controls for depression

A summary of the steps leading to the study sample is shown in Fig. S2 and the demographic characteristics of the final sample populations are shown in Table S1. Different sources of phenotypic information were used to define depression cases in the UK Biobank. Briefly, five measures for depression were identified and based on the number of measures endorsed by UK Biobank participants, one group of depression cases was created. Participants who met the criteria for at least two of the five depression measures were defined as depression cases. Using multiple phenotype sources is an approach to optimize the sample size of credible cases without increasing misclassification bias between cases and controls. It has been reported that using at least two or more measures to define depression cases provides a good approximation of depression [38].

The five depression measures that were identified are “help-seeking”, “self-reported depression”, “antidepressant usage”, “depression (Smith)” [39] and “hospital (ICD-10)”. These measures have been used in a previous study by Glanville et al. [38] and we created our cases and controls based on the information from that study. After identifying cases for each of the five measures, cases were stratified by the number of endorsed measures. However, we made an exception for one of the measures, the ICD-coded depression. Since ICD-10 coded depression cases from hospital records is regarded as one of the most reliable measures, all the ICD-10 coded cases were included in the analysis even if the ICD-10 measure was the only measure endorsed by participants. For all other measures, at least two measures were required to ascertain depression cases.

The participants who did not meet the criteria for any of the above mentioned five depression measures were identified as controls. Moreover, the participants who did not endorse the question “Have you been diagnosed with one or more of the following mental health problems by a professional, even if you don’t have it currently?” for depression in the Mental Health Questionnaire were also classified as controls. All the criteria and UK Biobank codes that were used in the definition of both cases and controls are provided in Section S1 and S2.

#### 2.3.2 Cases and controls for co-morbid traits

A summary of the steps leading to the study sample is shown in Fig. S2 and the demographic characteristics of the final sample populations are shown in Table S1. The cases and controls for three co-morbid traits including anxiety, bipolar disorder and schizoaffective disorders were identified using both self-reported data and ICD 10 codes. Participants were determined as cases if they either had any primary or secondary diagnosis of ICD-10 codes for anxiety (F40-F41), bipolar disorder (F30-F31) and/or schizoaffective disorder (F30-F31) from linked hospital admission records or if they had a self-reported non-cancer illness code for anxiety (code:1287), bipolar disorder (code:1291) or schizophrenia (code:1289) of the UK Biobank field ID (FID): 20002. Controls for all three co-morbid traits were participants who did not meet any criteria for anxiety, bipolar disorder or schizophrenia.

#### 2.3.3 Exclusion criteria

In the UK Biobank, phenotypic data was available for 502,717 participants. For both depression and co-morbid traits, we excluded participants who had withdrawn their consent (n = 174), were genetically related (UK Biobank FID: 22021; n = 150,338) or were non-European (UK Biobank FID: 21000; n = 59,928). After this general exclusion criteria, we further applied specific exclusion criteria for depression and co-morbid traits to reduce the overlap between the cases. For depression, all the participants diagnosed with anxiety, bipolar disorder or schizoaffective disorders were excluded. For co-morbid traits, all the participants identified as having depression were excluded (Fig. S2). Although this strategy may affect the potential to detect pleiotropic genes, our focus on individuals with only depression without comorbid traits or with comorbid traits without depression aims to reduce the overlap between the groups.

### 2.4 Statistical analyses

Each SNP has three genotype categories since each individual inherits two copies of chromosomes with one allele copy from each parent. The three genotype categories were coded as (0) for homozygous of major allele, (1) for heterozygous genotype, and (2) for homozygous of minor allele. To increase the sample size for the co-morbid traits, all the three phenotypes for anxiety, bipolar disorder and schizoaffective disorders were grouped together into one phenotype called “co-morbid traits”. Binary logistic regression models adjusted for age, sex and first 10 genetic principal components were used to analyze the association between each genetic variant and depression/co-morbid traits. A p-value of <0.001 (0.05/38) was deemed statistically significant after being adjusted for multiple testing using the Bonferroni correction. The p-value is derived from the analysis of the 38 tested SNPs, irrespective of the two phenotypes, given that depression and co-morbid traits represent distinct and independent datasets of participants.

After running the binary logistic regression models, the SNPs that were associated with each phenotype based on the significance level (p-value <0.001) were identified. All the SNPs were recoded to ensure uniform direction of effect with regard to the risk allele. In the next step, two genetic risk scores were created, a) the SNPs that were significantly associated with depression were summed up to create a genetic risk score for depression (GRS_dep-sig_), and b) another genetic risk score was also created based on all SNPs regardless of their significance level with depression (GRS_dep-all_). The two risk scores were used in binary logistic regression models to predict both depression and co-morbid phenotypes separately. The risk scores were used both as continuous variables as well as quartiles treated as numerical variables. Quartiles were created as a set of descriptive statistics to split the score into four equal parts, to compare the genetic load by each quartile. Furthermore, sex-stratified analyses were conducted to investigate the predictive power of genetic risk scores in men and women separately. We also performed tests for interactions between the risk scores and sex to further explore the influence of sex. All binary logistic regression models aimed at analyzing the association between each of the two risk scores and both the depression and co-morbid phenotypes were conducted using a nominal significance level for the p-value. The primary hypotheses centered around two key questions: 1) Is there an association with depression? and 2) Is there an association with co-morbid traits? Given that each of the depression and co-morbid phenotypes constituted distinct entities, with each encompassing its own independent dataset of participants, the utilization of multiple correction techniques was deemed non-essential. All statistical analyses were conducted using IBM SPSS Statistics version 26 (IBM SPSS, Armonk, NY, USA).

### 2.5 Transcriptome analyses

Transcriptome analyses were conducted for identified depression-related genes based on the results from the UK biobank data. The gene list included six genes: Dystroglycan 1 (*DAG1*), Fragile Histidine Triad Diadenosine Triphosphatase (*FHIT*), Butyrophilin Subfamily 3 Member A2 (*BTN3A2*), Tenascin XB (*TNXB*), Latent Transforming Growth Factor Beta Binding Protein 3 (*LTBP3*), and Neural Cell Adhesion Molecule 1 (*NCAM1*). For this analysis, we used three publicly-available cohorts (GSE53987 (n = 35), GSE98793 (n = 192), and GSE46743 (n = 160)) deposited in the Gene Expression Omnibus (GEO) [40]. The cohort GSE53987 contained expression data on several brain regions (hippocampus, pre-frontal cortex, associative striatum), whereas GSE98793 and GSE46743 contained similar data for the whole blood. In the cohort GSE53987, MDD-confirmed cases were compared against non-affected controls. In the cohorts GSE98793 GSE46743, MDD participants were compared to non-affected controls at baseline. We used limma R package to identify differentially expressed transcripts. All analyses were adjusted for potential confounding depending on available phenotypic data. Raw nominal p-values from limma (p < 0.05) were considered, while the FDR-adjusted p-values and family-wise error rates were additionally reported but not considered in the analysis. Detailed descriptions of cohorts, data processing and analysis are found in Section S3.

### 2.6 Methylation analyses

Methylation analyses were conducted to identify potential changes in methylation among depression-related genes. For this purpose, we used data from three independent cohorts (PSY-SCR, GSE72680, and GSE125105). The first cohort Psychiatric Health in Adolescents in Uppsala Cohort (PSY-SCR) is a local cohort in our research group and includes 221 non-related adolescents aged 14–16 that were recruited from public schools in Uppsala County, Sweden between 2012 and 2013. All participants gave their written informed consent regarding participation in the study and the study was approved by the Regional Ethics Committee of Uppsala. For further details about the cohort, please refer to our previous publications [41,42]. The other two cohorts were publicly available and could be found on GEO: GSE72680 (n = 312) and GSE125105 (n = 491). We used whole blood methylation data from all cohorts. The phenotypic characterization of the PSY cohort was performed with the Development and Well-Being Assessment (DAWBA) [43] questionnaire. Beck Depression Incentory (BDI) [44] was used in GSE72680, whereas clinical-like assessment has been performed in GSE125105. DNA methylation data passed through a series of preprocessing and filtering steps, and we used the limma R package to identify differentially methylated CpGs. All analyses were adjusted for potential confounding depending on the available phenotypic data. Raw nominal p-values from limma (p < 0.05) were considered, while the FDR-adjusted p-values and family-wise error rates were additionally reported but not considered in the analysis. Detailed information about the cohorts and extensive descriptions of methodology are found in Section S4.

## 3 Results

### 3.1 Association between common genetic variants and depression/co-morbid traits

The binary logistic regression models adjusted for age, sex and first ten genetic principal components identified eight SNPs that were significantly associated with depression (p-value <0.001 for all). Out of the eight significantly associated SNPs with depression, 2 pairs of SNPs corresponded to the same gene while each of the remaining SNPs corresponded to a specific gene (Table S2). No significant associations were observed between any of the SNPs and co-morbid traits (Table S3).

### 3.2 Association between genetic risk scores and depression/co-morbid traits

**Fig. 1:**
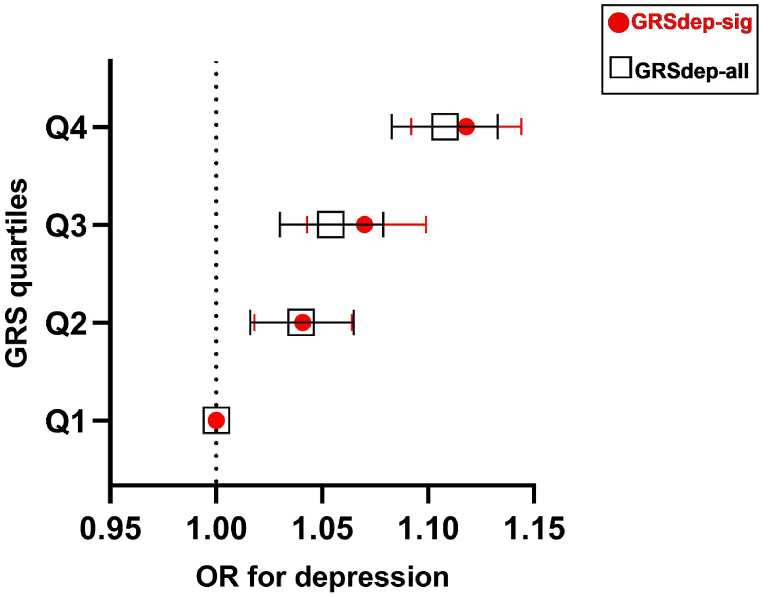
Forest plot for the association of genetic risk scores with depression. This figure shows the association of genetic risk scores with depression for both genetic risk scores; GRSdep-sig and GRSdep-all. The X-axis shows the OR for depression; the Y-axis corresponds to the GRS quartiles. Error bar symbols and colors indicate the following: red dot – GRSdep-sig; black rectangle – GRSdep-all. The figure was created using Graphpad Prism 9. Abbreviations: GRS, genetic risk score; GRSdep-sig, genetic risk score for significant genetic variants, GRSdep-all, genetic risk score for all genetic variants.

The GRS_dep-sig_ was significantly associated with depression [odds ratio (OR) (95 % confidence interval (CI)): 1.020 (1.016–1.023); p: 1.42 × 10^−24^] but not with co-morbid traits [OR (95 % CI): 1.003 (0.972–1.036); p: 0.844] as shown in Tables S4 and S5. Similarly, the GRS_dep-all_ was significantly associated with depression [OR (95 % CI): 1.009 (1.007–1.011); p: 7.47 × 10^−24^] but not with co-morbid traits [OR (95 % CI): 0.995 (0.980–1.010); p: 0.477] as shown in Tables S4 and S5. Moreover, participants in the highest GRS_dep-sig_ quartile had 11.8 % increased odds for depression [OR (95 % CI): 1.118 (1.092–1.144); p: 6.86 × 10^−21^] compared with participants in the lowest GRS_dep-sig_ quartile (Fig. 1 and Table S6). Similarly, participants in the highest GRS_dep-all_ quartile had 10.8 % increased odds for depression [OR (95 % CI): 1.108 (1.083–1.133); p: 4.27 × 10^−19^] compared with participants in the lowest GRS_dep-all_ quartile (Fig. 1 and Table S6). No significant interactions were observed between any of the risk scores and sex in any of the tests conducted.

### 3.3 Sex-stratified analysis for the association of genetic risk scores with depression and co-morbid traits

**Fig. 2:**
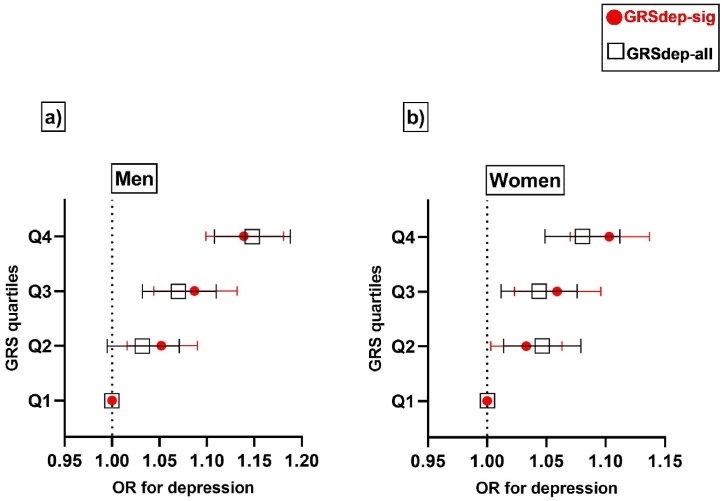
Forest plot for the sex-stratified association of genetic risk scores with depression. This figure shows the sex-stratified association of genetic risk scores with depression for a) men and B) women for both genetic risk scores; GRSdep-sig and GRSdep-all. The X-axis shows the OR for depression; the Y-axis corresponds to the GRS quartiles. Error bar symbols and colors indicate the following: red dot – GRSdep-sig; black rectangle – GRSdep-all. The figure was created using Graphpad Prism 9. Abbreviations: GRS, genetic risk score; GRSdep-sig, genetic risk score for significant genetic variants, GRSdep-all, genetic risk score for all genetic variants.

In the sex-stratified analyses, the GRS_dep-sig_ was significantly associated with increased risk for depression in both men [OR (95 % CI): 1.023 (1.017–1.029); p: 1.41 × 10^−14^] and women [OR (95 % CI): 1.017 (1.012–1.022); p: 5.70 × 10^−12^] (Table S7). Similarly, the GRS_dep-all_ was significantly associated with increased risk for depression in men [OR (95 % CI): 1.011 (1.009–1.014); p: 1.26 × 10^−16^] and women [OR (95 % CI): 1.007 (1.005–1.009); p: 6.09 × 10^−10^] (Table S7). No significant associations were observed for GRS_dep-sig_ or GRS_dep-all_ with co-morbid traits in either men or women (p-value >0.001 for all) (Table S5 and TableS8-S10). Furthermore, men in the highest GRS_dep-sig_ quartile had 13.9 % increased odds for depression [OR (95 % CI): 1.139 (1.099–1.181); p: 1.30 × 10^−12^] and men in the highest GRS_dep-all_ quartile had 14.8 % increased odds for depression [OR (95 % CI): 1.148 (1.108–1.188); p: 8.44^−15^] as compared to participants in their respective lowest quartiles (Fig. 2 and Table S11). Similarly, women in the highest GRS_dep-sig_ quartile had 10.3 % increased odds for depression [OR (95 % CI): 1.103 (1.070–1.137); p: 3.37 × 10^−10^] and women in the highest GRS_dep-all_ quartile had 8 % increased odds for depression [OR (95 % CI): 1.080 (1.049–1.112); p: 3.0110^−7^] as compared to participants in the lowest quartiles of GRS_dep-sig_ and GRS_dep-all_ respectively (Fig. 2 and Table S11).

### 3.4 Transcriptome analysis

We sought to explain the associations between identified SNPs, their associated genes, and depression. For this task, we performed differential expression analyses of probes from six depression-associated genes from UK-biobank data in three publicly available cohorts (GSE53987 (n = 35), GSE98793 (n = 192), and GSE46743 (n = 160). Phenotypical characterization of the cohorts could be found in Section S5. Each cohort was stratified into two groups based on depression diagnosis, depressed or non-depressed (see methods). The cohort GSE53987 was nearly identical in all characteristics in depressed and non-depressed groups. In the GSE98793, there was no difference between groups as well except a relatively higher proportion of women than men (75 % vs 25 %). For the GSE46743 cohort, the age of participants was relatively higher for depressed group.

Transcriptome profiling of gene-related probes did not show fully overlapping results, and no results were significant after the adjustment for multiple comparisons (Table S13). However, we could observe that some TNXB-related probes show a nominal statistically significant association (not corrected for multiple comparisons) with depressed phenotype. The direction of association is consistent within the first cohort (GSE53987) across the hippocampus, pre-frontal cortex (*BA46*), and associative striatum, but does not overlap with GSE98793. Additionally, *NCAM1*-related probes were associated with depression in both GSE98793 and GSE46743 cohorts but the direction of the association is not concordant. Moreover, the absolute changes in the transcript concentrations were small for all the investigated probes.

### 3.5 Methylation analysis

Similarly, we investigated identified genes at the epigenetic level, conducting a differential methylation analysis for relevant CpG sites. We attempted to make the analysis consistent and used available cohorts that have methylation data coming from the same array (HumanMethylation450). In total, we used three independent cohorts (two of which are public) to identify cross-validated differentially methylated CpG sites. Each of the cohorts was split into two subgroups regarding depression risk/diagnosis and non-affected controls. Characteristics of the cohorts are provided in Section S6. In the adolescent cohort, the high depression risk group was enriched with women (>90 %). Other cohorts (GSE72680 and GSE125105) were relatively similar in all characteristics, with the exception of treatment data in GSE72680 (Section S6).

**Fig. 3:**
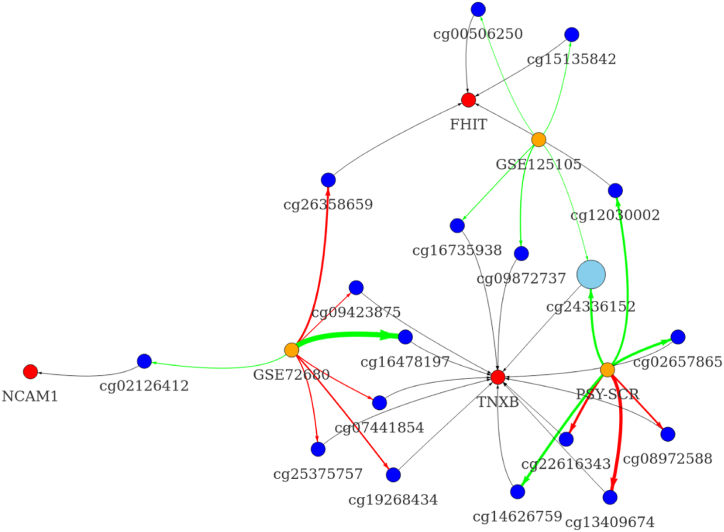
Differential methylation analysis graph of promoter probes. This figure shows nominally significant results for differential methylation analysis of promoter-associated probes in three investigated cohorts. Node colors indicate the following: red – gene, blue – methylation site, light-blue – cross-significant methylation site, and orange – the cohort used. Edge color corresponds to the direction of the association, where green indicates downregulation and red – upregulation. The thickness of the edge is proportional to the effect size (absolute of log2 fold change) for a specific probe. Edges between a probe and associated gene are shown in black and thickness is constant. The image was created using the “visNetwork” R package. Abbreviations: NCAM1, neural cell adhesion molecule 1; FHIT, fragile histidine triad diadenosine triphosphatase; TNXB, tenascin XB.

**Fig. 4:**
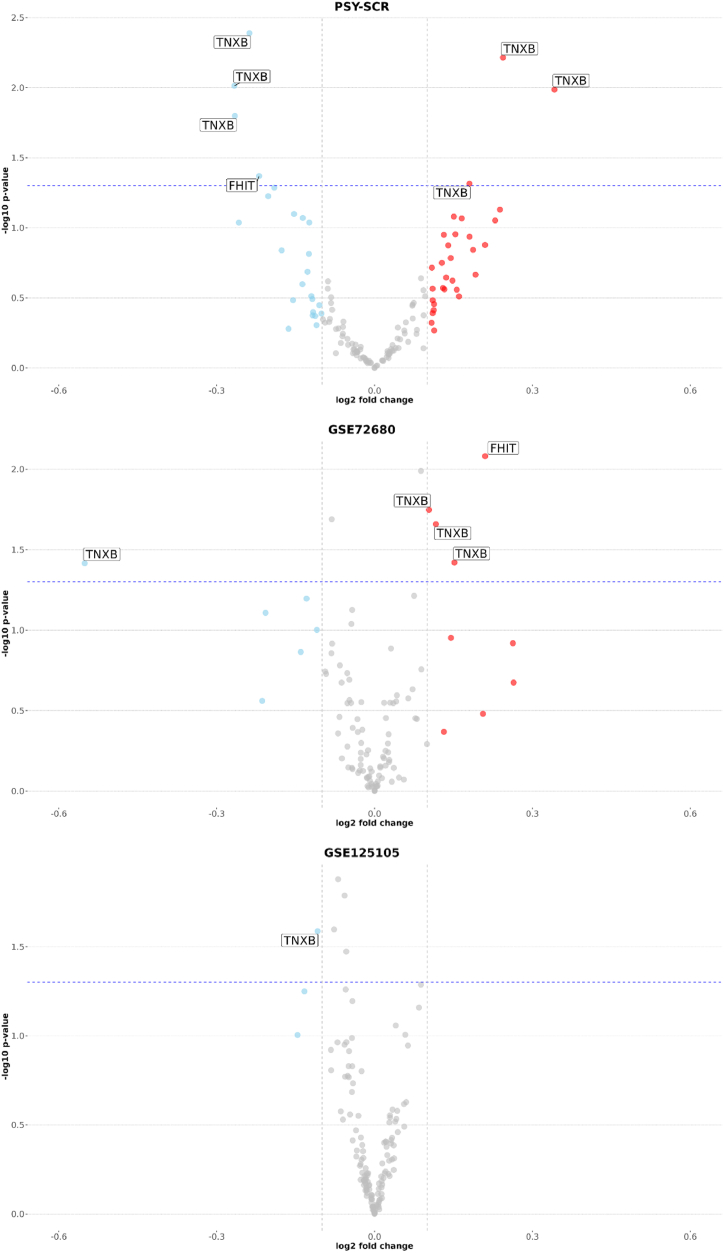
Volcano plots for differential methylation analysis (promoter region). This figure shows volcano plots for differential methylation analysis of promoter-related CpGs in all three cohorts. The X-axis shows log2 fold change; the Y-axis corresponds to -log10 p-value. The blue dashed line indicates the threshold for nominal significance. Gray vertical lines indicate −0,1 and 0,1 thresholds for log2 fold change estimations. Dots in color indicate probes that have passed log2 fold change thresholds. Gene names are shown for probes that passed log2 fold change thresholds and nominal significance thresholds. This figure corresponds to data in Table S14.

**Fig. 5:**
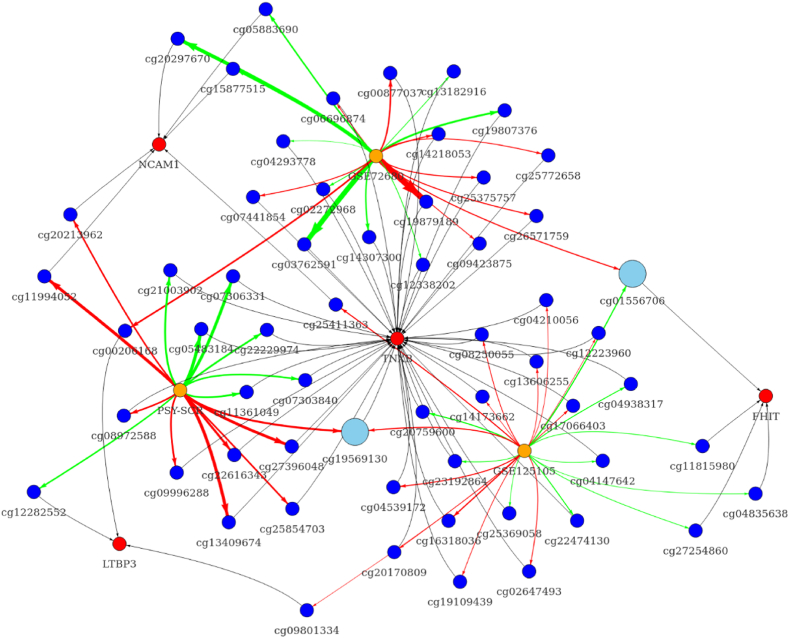
Differential methylation analysis graph of gene body CpGs. This figure shows nominally significant results for differential methylation analysis of gene-body-associated probes in three investigated cohorts. Node colors indicate the following: red – gene, blue – methylation site, light-blue – cross-significant methylation site, and orange – the cohort used. Edge color corresponds to the direction of the association, where green indicates downregulation and red – upregulation. The thickness of the edge is proportional to the effect size (absolute of log2 fold change) for a specific probe. Edges between a probe and associated gene are shown in black and thickness is constant. The image was created using the “visNetwork” R package. Abbreviations: NCAM1, neural cell adhesion molecule 1; FHIT, fragile histidine triad diadenosine triphosphatase; TNXB, tenascin XB; LTBP3, Latent Transforming Growth Factor Beta Binding Protein 3.

**Fig. 6:**
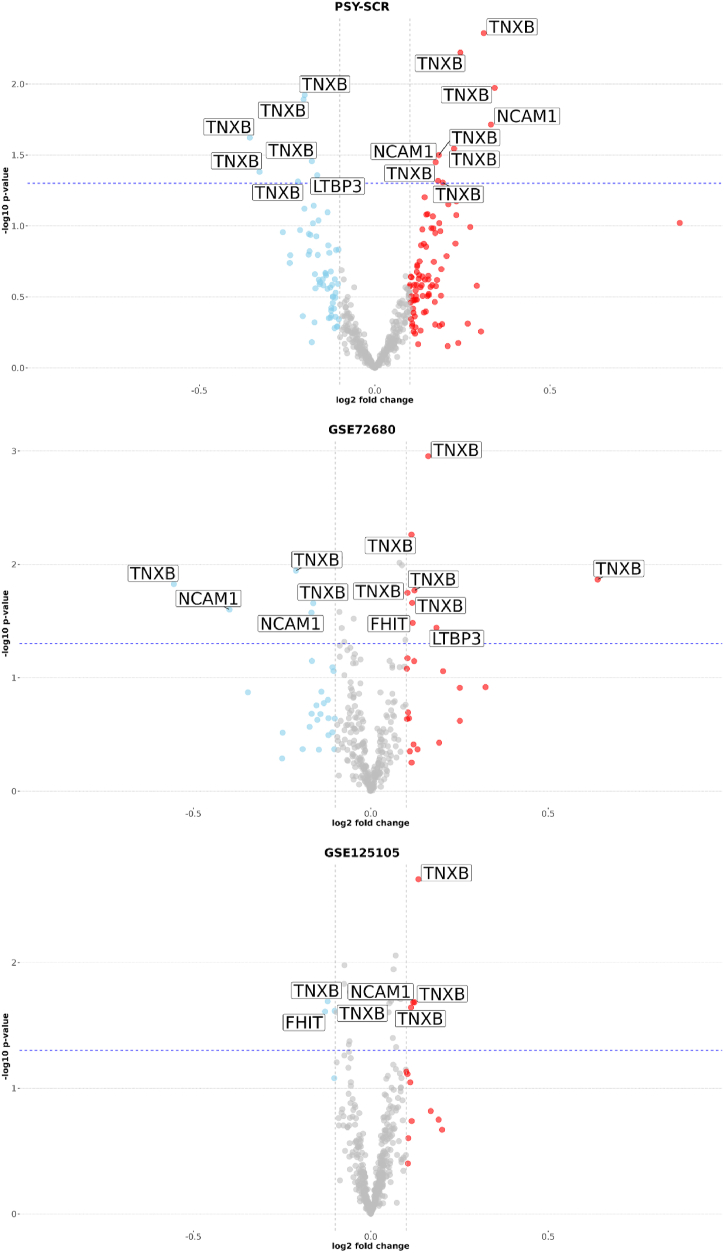
Volcano plots for differential methylation analysis (gene body region). This figure shows volcano plots for differential methylation analysis of gene-body-associated CpGs in all three cohorts. The X-axis shows log2 fold change; the Y-axis corresponds to -log10 p-value. The blue dashed line indicates the threshold for nominal significance. Gray vertical lines indicate −0,1 and 0,1 thresholds for log2 fold change estimations. Dots in color indicate probes that have passed log2 fold change thresholds. Gene names are shown for probes that passed log2 fold change thresholds and nominal significance thresholds. This figure corresponds to data in Table S15.

Differential methylation analysis identified several nominally-significant associations for promoter and gene body CpGs, however none of them passed the adjustment for multiple comparisons (Tables S14–S15). In the promoter regions, we identified one TNXB-related probe (cg24336152) that showed decreased methylation in the depressed group in more than one cohort (Fig. 3 and Table S14). Other probes did not show overlap among different studies. Interestingly, the effect sizes for probes were associated with a corresponding cohort, and all probes in GSE125105 showed small differences in methylation between cases and controls (Fig. 4). A similar analysis of gene body-located methylation sites yielded two nominally-significant differentially regulated CpGs. However, only one of these probes (cg19569130, TNXB) showed a consistent increase in methylation in more than one cohort (Fig. 5, Fig. 6; Table S15). Similarly, gene body-located probes in GSE125105 demonstrated minuscule differences for analyzed groups. None of the investigated probes were cross-validated in all the three cohorts.

## 4 Discussion

The current study aimed to identify independent genetic variants in protein-coding genes that associate with depression to provide the foundation for prediction tool to enhance personalized treatment of depression. We combined the genetic variants after validation in the UK Biobank cohort into a genetic risk score for depression and examined the association with depression and other co-morbid traits to evaluate its predictive ability and identified eight SNPs that were highly associated with depression corresponding to six protein-coding genes (*DAG1, FHIT, BTN3A2, TNXB, LTBP3, NCAM1*). We also used publicly available cohorts and conducted both transcriptomic and methylation analyses for the genes corresponding to the validated variants to explore differentially expressed transcripts and DNA methylation between depressed cases and healthy controls. Our transcriptome analysis showed only small changes in the transcript concentrations for investigated probes but we could observe nominal significance for some gene probes (*TNXB*- and *NCAM1*) with depressed phenotype. Similarly, our differential methylation analysis of promoter regions and gene body-located methylation sites showed only small differences in methylation between cases and controls. However, there were only few nominally significant differentially regulated CpG sites and these were associated with *TNXB*. The genetic risk score that we created for depression based on the identified SNPs was highly specific for depression and did not show a significant association with other co-morbid traits.

For the depression risk score, we compared the highest quartile with the lowest quartile to capture the genetic prediction by the combined set of genetic variants. The fact that participants in the upper quartiles have significantly increased risk and in some cases the risk is multifold as compared to those in the lowest quartile indicates that our risk score is able to capture the genetic load and might be helpful in providing individual risk estimation. Moreover, the risk score based on our validated genetic variants could differentiate between depression and other co-morbid traits since it was highly specific for depression and could not predict other co-morbid traits. This may be explained by the proper classification of the depression phenotype with minimal overlap with co-morbid cases. Interestingly, however, it was shown in some previous GWAS that the genetic risk for co-morbid traits such as bipolar disorder and schizophrenia is associated with depression or vice versa which was explained by the genetic overlap between the disorders [45,46]. Furthermore, while the tests for interactions between the risk scores and sex yielded non-significant results, our sex-stratified analyses revealed that the genetic risk score predicted higher risk for depression in men which is in line with the evidence that psychiatric disorders exhibit sex differences between men and women [47]. Although the exact mechanisms are still lacking, hormonal factors are known to influence gene expression which may lead to different responses to social stressors in men and women [48,49]. However, while the sex-stratified analyses could be suggestive of potential sex-specific effect, caution is needed when interpreting the sex-specific findings due to the non-significant interactions suggesting that the observed differences between the sexes might be due to random chance or other factors unrelated to the interaction between the risk score and sex.

Of the candidate genes that were associated with depression, *TNXB* attracted particular attention since it was the only gene that additionally showed nominal significance in both transcriptome and methylation analyses. *TNXB* has previously been shown to be differentially methylated in depression [50], and also to have altered transcriptomic profile in fibroblasts of depressive patients [51]. *TNXB* encodes Tenascin X, which is an extracellular matrix glycoprotein expressed in connective tissues with importance for both cell adhesion and cell motility [52]. Loss of the Tenascin X protein caused by mutations in the *TNXB* gene is associated with the development of the connective tissue disorder known as Ehlers-Danlos syndrome [53] and patients with Ehlers-Danlos syndrome have a more than threefold increased risk for depression [54]. The location of *TNXB* on chromosome 6 is within the extended major histocompatibility complex (MHC) class III region [55]. Many genes in or around MHC are essential for the regulation of the immune system and have been associated with an increased risk of autoimmune and inflammatory diseases [[56], [57], [58]]. There is well-established evidence for the involvement of the immune system in psychiatric illness [59]. Recent findings from GWAS studies have also reported genetic variants in the MHC region associated with depression risk [10,60,61]. Our finding of *TNXB* is consistent with previous evidence to support the role of inflammation in the pathophysiology of depression [62,63].

Another interesting finding is the *NCAM1* gene, also known as CD56, which is a member of the immunoglobulin superfamily [64]. *NCAM1* is located on chromosome 11q and has a modulatory function in intracellular cascades and brain plasticity [65]. Numerous studies have assumed *NCAM1* as a susceptibility factor for several neuropsychiatric disorders, including depression [[66], [67], [68]], bipolar disorder [69,70], and schizophrenia [71,72]. Additionally, several genetic variants in the *NCAM1* gene have been linked to an increased risk of the development of bipolar disorder [73], schizophrenia [74], autism [75], as well as other conditions, such as suicidal behavior [76] and cannabis use and depression [77]. Moreover, experimental studies have shown deficits in *NCAM1* to be associated with depression-like phenotype and reduced antidepressant efficacy in animal models. Given the aforementioned roles and its critical role in neural and synaptic plasticity [[78], [79], [80], [81], [82], [83]], *NCAM1* is highly interesting as a potential candidate gene.

Other potential candidate genes whose function might provide new insights in the pathophysiological process of depression include *LTBP3, BTN3A2, DAG1* and FHIT*. LTBP3* is an extracellular matrix protein which is suggested to be involved in the transforming growth factor beta (TGFβ) signaling [84,85]. *TGF-β* pathways, which in turn, are involved in mediating several aspects of neuroplasticity events have been implicated in neuropsychiatric manifestations [86]. Genetic correlation studies have found that genetic variants in the *TGF-β* family of proteins were associated with antidepressant treatment outcome [87]. *BTN3A2* is located in the MHC region [88], and *t*he *BTN3A2* protein is suggested to be involved in adaptive immune response [89]. *BTN3A2* is expressed in multiple cell types in the brain, including astrocyte, neuron, oligodendrocyte, and microglia [90]. Previous studies have implicated the role of *BTN3A2* as a potential risk gene for Alzheimer's disease, schizophrenia, and intellectual disability [[91], [92], [93]]. Additionally, a meta-analysis of GWAS has indicated the association of *BTN3A2* with neuroticism [94], which is an important risk factor for depression [95]. *DAG1* is a cytoskeleton-linked extracellular matrix receptor composed of α- and β-subunits with several functions including cell survival, peripheral nerve myelination, nodal structure and cell migration [96]. Variants in *DAG1* have been associated with both type A and type C muscular dystrophy–dystroglycanopathy [[97], [98], [99], [100]]. Some findings have demonstrated the role of *DAG1* in the pathophysiological process of depressive-like behaviors by regulating GABAergic neurotransmission [101]. Finally, the *FHIT* gene is a tumor suppressor protein involved in multiple cancers [102]. It is also a circadian clock modifier gene [103] and has been linked with daytime sleepiness [104], which may be relevant for depression. *FHIT* is expressed in several brain regions including the amygdala, anterior cingulate cortex, caudate nucleus, prefrontal cortex, hippocampus and hypothalamus [105]. Genetic variants within the *FHIT gene* have been implicated in anxiety [106], comorbid depressive syndromes and alcohol dependence [107], mental stress [108] and autism [109]. In addition, a recent GWAS meta-analysis has identified a new locus for depression within the *FHIT* gene [110].

Finally, transcriptome and methylation analyses indicated a very weak connection of *TNXB* with depressive phenotypes. However, these results could be considered non-significant after adjustment for multiple comparisons that warrant further investigation. Overall, our findings provide new insights into different potential mechanisms underlying depression and present new clues for future research. Although individual genes may have little impact, a genetic risk scores based on a combination of genes can be used as a tool for genetic risk stratification that would assist decisions about interventions during the early stages of psychiatric disease such as participation in screening programs and lifestyle modifications. Although genetic risk scores as we have created in the current study alone may not capture the full genetic loading for a disorder, it may at least capture part of an individual's susceptibility to depression and its predictive value becomes higher along with other risk factors. Future research should focus on multicomponent risk prediction models to efficiently identify individuals with elevated risk. Psychiatry needs a combined set of known factors, such as biochemistry, lifestyle, historical and clinical risk factors in the same way as those currently used to predict risk for cardiovascular disease [111] and breast cancer [61].

The current study has both strengths and limitations. One strength is our large sample size based on the UK Biobank which is one of the largest population-based cohorts. Secondly, we used multiple measures of depression with strict inclusion and exclusion criteria to increase the liability of cases and reduce misclassification. Moreover, the fact that cases were partly identified through clinical records increases the relevance for clinical practice. Thirdly, we used GWAS Catalog, a curated database [112] to identify our independent genetic variants for depression for further validation in the UK Biobank cohort. We selected genes that were measurable by proteomic techniques which enables further analyses of our results. Our study can be regarded as a survey of depression-associated variants which offers a selection of interesting loci that should be focused on in future studies.

To mention some limitations, we included only intragenic variants from GWAS Catalog as we were interested in variants located within protein-coding genes that can be measured by Olink's Proteomics, to facilitate proteomic studies on these genes in future research. The implementation of this strategy limited the selection of variants and reduced the probability of detecting intergenic variants. Another limitation relates to the selection of depression-specific SNPs using the GWAS Catalog and the use of UKB as a validation cohort. Some of the resulting 38 SNPs may have already been identified in genetic association studies that utilized UKB data. Consequently, it would be valuable to validate these SNPs in an independent cohort. The same applies to the GRSs, which were created and tested within the UKB. For a better evaluation of their predictive abilities, it would be valuable to test these GRSs in independent cohorts. In addition, it is also worth noting that grouping the three comorbid traits to increase the sample size is another concern. While there may be shared etiology and pathology among psychiatric comorbidities [113,114], schizophrenia, bipolar disorder, and anxiety, remain three distinct disorders that should be treated separately.

More limitations include the definition of cases that was partly based on self-reported data which may slightly reduce the liability of cases. The prevalence of depression in UKB is considerably higher than the general population due to less strict criteria for case definition, as well as and the exclusion of large numbers of controls due to incomplete or missing data. Moreover, the current study is based on data from European participants which may limit the generalization of the findings to other populations and ancestries. Another challenging aspect is moving genetic risk scores from research studies to clinical implementation as the genetic risk scores require cautious interpretations since they are only descriptive and represent a summary measure of genetic risk but do not inform any biological mechanism. The cohorts used in transcriptome and DNA methylation analyses may not be genetically similar to the UK biobank population, as they had small sample sizes and used different methods for definition of depression and data preparation, thus limiting comparability of the results. Also, the observed associations in DNA methylation and transcriptome were only nominal and non-significant after adjustment for multiple comparisons, thus requiring replication in other cohorts to confirm or refute these findings.

## 5 Conclusions

We have identified six protein-coding genes associated with depression and primarily involved in inflammation (*TNXB*), neuroplasticity (*NCAM1 and LTBP3),* immune response (*BTN3A2),* cell survival (*DAG1) and* circadian clock modification (*FHIT)*. Our findings confirmed previous evidence for *TNXB*- and *NCAM1* in the pathophysiology of depression and suggested four new potential candidate genes (*LTBP3, BTN3A2, DAG1* and FHIT*)* that warrant further investigation.

## CRediT authorship contribution statement

**Muataz S. Lafta:** Writing – original draft, Methodology, Formal analysis, Data curation, Conceptualization. **Aleksandr V. Sokolov:** Writing – review & editing, Validation, Data curation, Conceptualization. **Gull Rukh:** Writing – review & editing, Supervision, Data curation, Conceptualization. **Helgi B. Schiöth:** Writing – review & editing, Supervision, Resources, Conceptualization.

## Data availability

The data used in this current study is available from the UK Biobank Resource (https://www.ukbiobank.ac.uk). Permissions are required in order to gain access to the data, which was used under license for this study.

## Funding

Helgi Schiöth was supported by the 10.13039/501100004359Swedish Research Council (Vetenskapsrådet) and Swedish Brain Research Foundation. Gull Rukh was supported by the 10.13039/501100003748Swedish Society for Medical Research (Svenska Sällskapet för Medicinsk Forskning). Funders had no role in the design of the study, collection, analysis or in the writing the manuscript.

## Declaration of competing interest

All authors declare no conflict of interest.
