# Supplementary material for: Identification and validation of depression-associated genetic variants in the UK Biobank cohort with transcriptome and DNA methylation analyses in independent cohorts
Source: Heliyon. 2025 Jan 10;11(2):e41865. doi: 10.1016/j.heliyon.2025.e41865 (PMC11787470; doi:10.1016/j.heliyon.2025.e41865)
Supplement: Multimedia component 1 [file mmc1.docx]

Supplementary 1

Contents

[Figure S1: Flowchart genotypes 2](#_Toc132449536)

[Figure S2: Flowchart phenotypes 3](#_Toc132449537)

[TABLE S1: Demographic characteristics of the final sample populations of depression and comorbid traits 4](#_Toc132449538)

[TABLE S2: Binary regression analysis of the association between SNPs and depression phenotype. 4](#_Toc132449539)

[TABLE S3: Binary regression analysis of the association between SNPs and co-morbid traits. 7](#_Toc132449540)

[TABLE S4: Binary regression analysis of the association between genetic risk score for depression (GRSdep) and depression phenotype. 9](#_Toc132449541)

[TABLE S5: Binary regression analysis of the association between genetic risk score for depression (GRSdep) and co-morbid traits. 9](#_Toc132449542)

[TABLE S6: Binary regression analysis of the association between quartiles of genetic risk score for depression (GRSdep) and depression phenotype. 10](#_Toc132449543)

[TABLE S7: Sex-stratified binary regression analysis of the association between genetic risk score for depression (GRSdep) and depression phenotype. 10](#_Toc132449544)

[TABLE S8: Sex-stratified binary regression analysis of the association between genetic risk score for depression (GRSdep) and co-morbid traits. 11](#_Toc132449545)

[TABLE S9: Binary regression analysis of the association between quartiles of genetic risk score for depression (GRSdep) and co-morbid traits. 11](#_Toc132449546)

[TABLE S10: Sex-stratified binary regression analysis of the association between quartiles of genetic risk score for depression (GRSdep) and co-morbid traits. 12](#_Toc132449547)

[TABLE S11: Sex-stratified binary regression analysis of the association between quartiles of genetic risk score for depression (GRSdep) and depression phenotype. 12](#_Toc132449548)

[Section S1: Definition of depression cases and controls 13](#_Toc132449549)

[Section S2: Definition of co-morbid cases and controls 16](#_Toc132449550)

[Section S3: Transcriptome analyses 17](#_Toc132449551)

[Section S4: DNA methylation analyses 19](#_Toc132449552)

[Section S5: Transcriptomic cohorts 22](#_Toc132449553)

[Section S6: Methylation cohorts 25](#_Toc132449554)

[Section S7: References 27](#_Toc132449555)

# Figure S1: Flowchart genotypes

Flow chart showing the steps for the generation of the genotype data.


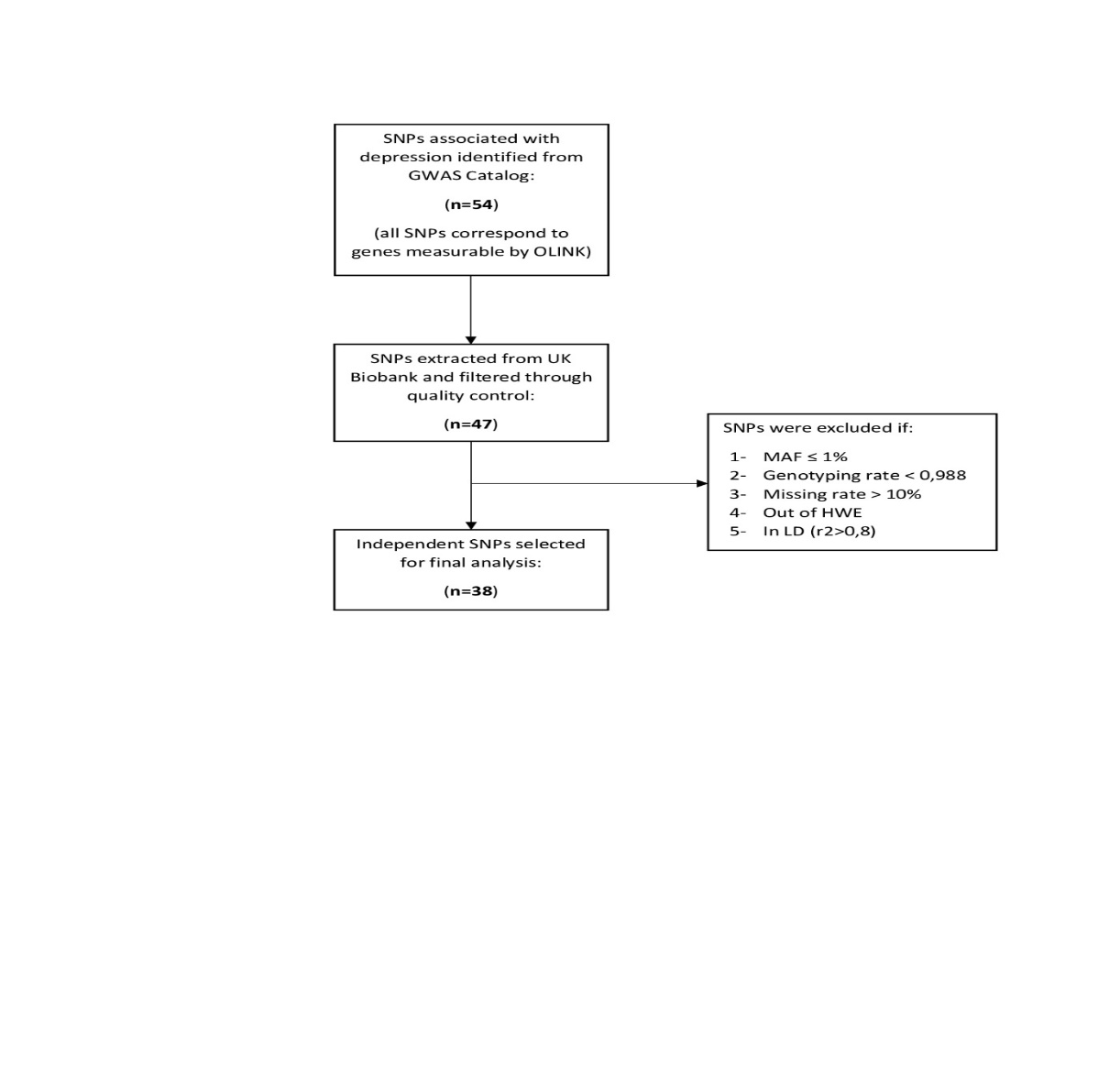


# Figure S2: Flowchart phenotypes

Flow chart showing the selection of study samples for depression and co-morbid traits in the UK biobank cohort.


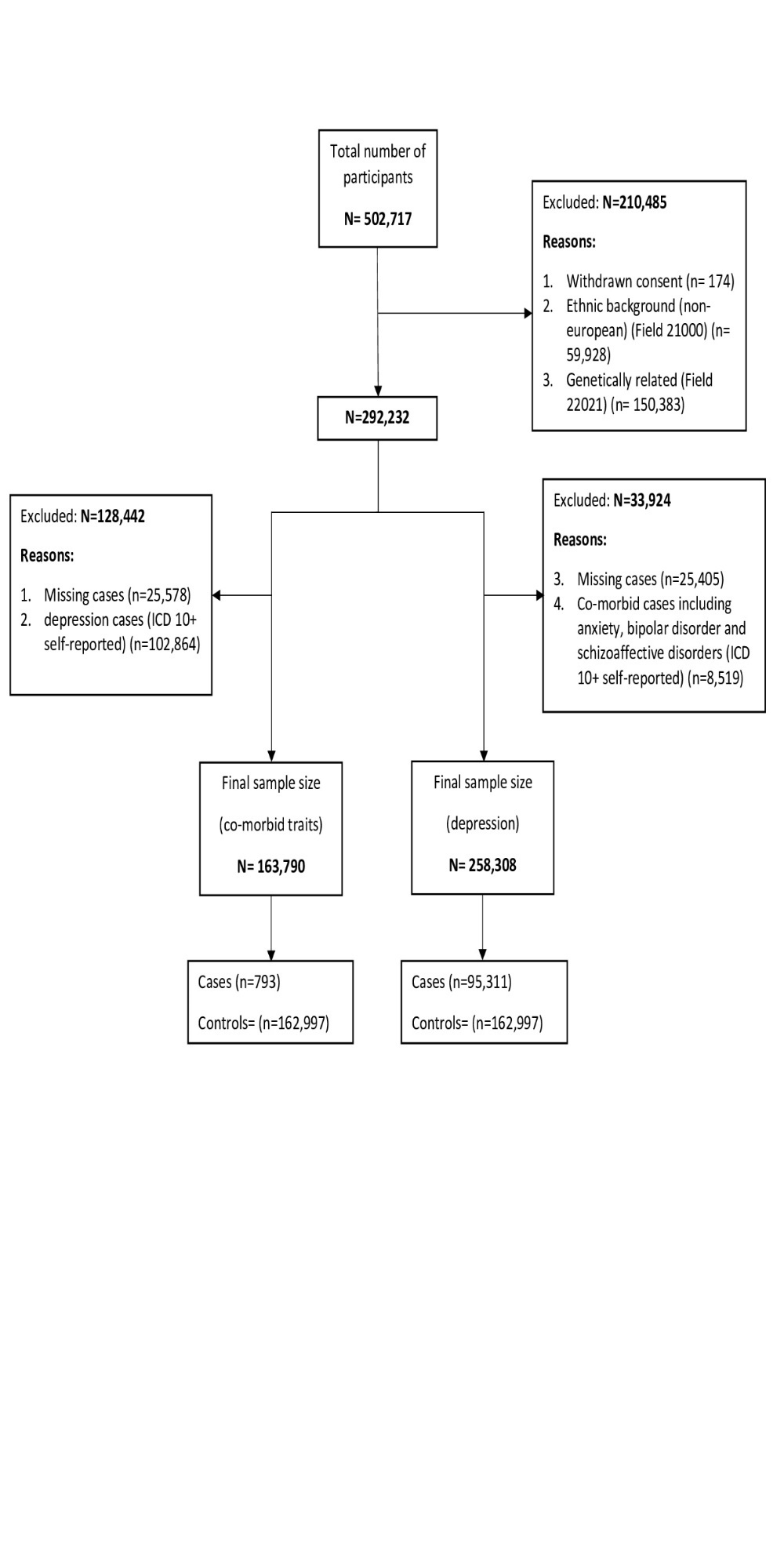


#

# TABLE S1: Demographic characteristics of the final sample populations of depression and comorbid traits

| **Depression** | | | |
| --- | --- | --- | --- |
| Variable | | Patients n = 95 311 | Controls n = 162 997 |
| Age (mean ± SD) | | 58 ± 7.8 | 57 ± 8.1 |
| BMI (mean ± SD) | | 27.6 ± 5.1 | 27.2 ± 4.5 |
| Sex (female/male), n (%) | | 61 219/34 092 (64/36%) | 76 137/86 860 (47/53%) |
| Antidepressant use | Yes | 7 992 (8.4%) | 44 (0.0%) |
|  | No | 68 147 (71.5%) | 110 778 (68.0%) |
|  | Missing | 19 172 (20.1%) | 52 175 (32.0%) |
| Current tobacco smoking | Yes, on most or all days | 8 816 (9.2%) | 10 173 (6.2%) |
|  | Only occasionally | 2 754(2.9%) | 6 963 (2.4%) |
|  | No | 83 680 (87.8%) | 148 798 (91.3%) |
|  | Prefer not to answer | 61 (0.1%) | 63 (0.0%) |
| Alcohol intake frequency | Daily or almost daily | 19 886 (20.9%) | 36 550 (22.4%) |
|  | Three or four times a week | 20 993 (22.0%) | 41 869 (25.7%) |
|  | Once or twice a week | 23 433 (24.6%) | 44 003 (27.0%) |
|  | One or three times a month | 11 648 (12.2%) | 16 807 (10.3%) |
|  | Special occasions only | 11 909 (12.5%) | 14 805 (9.1%) |
|  | Never | 7 360 (7.7%) | 8 861 (5.4%) |
|  | Prefer not to answer | 82 (0.1%) | 102 (0.1%) |
| **Comorbid traits** | | | |
| Variable | | Patients n = 793 | Controls n = 162 997 |
| Age (mean ± SD) | | 58 ± 8,0 | 57 ± 8,1 |
| BMI (mean ± SD) | | 27,9 ± 5,0 | 27,2 ± 4,5 |
| Sex (female/male), n (%) | | 406/387 (51/49%) | 76 137/86 860 (47/53%) |
| Antidepressant use | Yes | 1 (0.1%) | 44 (0.0%) |
|  | No | 638 (80.5%) | 110 778 (68.0%) |
|  | Missing | 154 (19.4%) | 52 175 (32.0%) |
| Current tobacco smoking | Yes, on most or all days | 94 (11.9%) | 10 173 (6.2%) |
|  | Only occasionally | 9 (1.1%) | 3 963 (2.4%) |
|  | No | 689 (86.9%) | 148 798 (91.3%) |
|  | Prefer not to answer | 1 (0.1%) | 63 (0.0%) |
| Alcohol intake frequency | Daily or almost daily | 158 (19.9%) | 36 550 (22.4%) |
|  | Three or four times a week | 169 (21.3%) | 41 869 (25.7%) |
|  | Once or twice a week | 212 (26.7%) | 44 003 (27.0%) |
|  | One or three times a month | 71 (9.0%) | 16 807 (10.3%) |
|  | Special occasions only | 105 (13.2%) | 14 805 (9.1%) |
|  | Never | 76 (9.6%) | 8 861 (5.4%) |
|  | Prefer not to answer | 2 (0.3%) | 102 (0.1%) |

TABLE S2: Binary regression analysis of the association between SNPs and depression phenotype.

Analysis was adjusted for age, sex and first ten genetic principal components. P-values marked with bold indicate statistically significant p-values (p-value<0,05/38=0,001).

|  |  | | | | | | | | | |
| --- | --- | --- | --- | --- | --- | --- | --- | --- | --- | --- |
| SNP | | Odds ratio | Lower CI | Upper CI | P-value | Effect allele | Associated genes | CHR | Pubmed-ID | Link |
| rs10746509 | | 0,985 | 0,974 | 0,997 | 1,40E-02 | T | GALNT2 | 1 | 30643256 | www.ncbi.nlm.nih.gov/pubmed/30643256 |
| rs2042772 | | 1,006 | 0,993 | 1,019 | 3,91E-01 | T | LY75-CD302, LY75 | 2 | 27479909 | www.ncbi.nlm.nih.gov/pubmed/27479909 |
| rs7374731 | | 1,031 | 1,018 | 1,045 | **4,94E-06** | C | DAG1 | 3 | 30643256 | www.ncbi.nlm.nih.gov/pubmed/30643256 |
| rs4625 | | 1,010 | 0,997 | 1,022 | 1,35E-01 | G | DAG1 | 3 | 29942085 | www.ncbi.nlm.nih.gov/pubmed/29942085 |
| rs9825823 | | 0,990 | 0,978 | 1,001 | 7,36E-02 | T | FHIT | 3 | 28049566 | www.ncbi.nlm.nih.gov/pubmed/28049566 |
| rs141954845 | | 0,970 | 0,959 | 0,982 | **4,30E-07** | A | FHIT | 3 | 30718901 | www.ncbi.nlm.nih.gov/pubmed/30718901 |
| rs7432943 | | 1,031 | 1,019 | 1,043 | **3,09E-07** | C | FHIT | 3 | 34045744 | www.ncbi.nlm.nih.gov/pubmed/34045744 |
| rs935526 | | 1,011 | 0,999 | 1,023 | 7,40E-02 | C | ROBO2 | 3 | 29942085 | www.ncbi.nlm.nih.gov/pubmed/29942085 |
| rs775766 | | 0,995 | 0,982 | 1,008 | 4,25E-01 | A | ROBO2 | 3 | 29942085 | www.ncbi.nlm.nih.gov/pubmed/29942085 |
| rs114216628 | | 1,017 | 0,962 | 1,075 | 5,50E-01 | T | ROBO1 | 3 | 34782712 | www.ncbi.nlm.nih.gov/pubmed/34782712 |
| rs17382228 | | 0,994 | 0,981 | 1,006 | 3,36E-01 | G | SORCS2 | 4 | 29160301 | www.ncbi.nlm.nih.gov/pubmed/29160301 |
| rs6822806 | | 1,010 | 0,992 | 1,028 | 2,74E-01 | C | UNC5C | 4 | 29317602 | www.ncbi.nlm.nih.gov/pubmed/29317602 |
| rs74378198 | | 0,978 | 0,948 | 1,008 | 1,49E-01 | G | OSMR | 5 | 25897834 | www.ncbi.nlm.nih.gov/pubmed/25897834 |
| rs10514108 | | 1,002 | 0,983 | 1,022 | 8,21E-01 | T | ARSB | 5 | 30468137 | www.ncbi.nlm.nih.gov/pubmed/30468137 |
| rs58825580 | | 0,960 | 0,944 | 0,976 | **1,13E-06** | T | BTN3A2 | 6 | 34634379 | www.ncbi.nlm.nih.gov/pubmed/34634379 |
| rs13218591 | | 0,973 | 0,961 | 0,986 | **2,33E-05** | T | BTN3A2 | 6 | 29942085 | www.ncbi.nlm.nih.gov/pubmed/29942085 |
| rs41316748 | | 0,957 | 0,930 | 0,985 | 2,82E-03 | C | TNXB | 6 | 30643256 | www.ncbi.nlm.nih.gov/pubmed/30643256 |
| rs1150757 | | 1,035 | 1,018 | 1,053 | **7,56E-05** | A | TNXB | 6 | 30643256 | www.ncbi.nlm.nih.gov/pubmed/30643256 |
| rs2269426 | | 0,982 | 0,970 | 0,994 | 2,45E-03 | A | TNXB | 6 | 30643256 | www.ncbi.nlm.nih.gov/pubmed/30643256 |
| rs116078874 | | 1,011 | 0,969 | 1,054 | 6,18E-01 | A | GLO1 | 6 | 29317602 | www.ncbi.nlm.nih.gov/pubmed/29317602 |
| rs3823624 | | 0,976 | 0,962 | 0,991 | 1,65E-03 | T | MAD1L1 | 7 | 30718901 | www.ncbi.nlm.nih.gov/pubmed/30718901 |
| rs144631932 | | 0,985 | 0,940 | 1,031 | 5,13E-01 | G | ENPP2 | 8 | 33483693 | www.ncbi.nlm.nih.gov/pubmed/33483693 |
| rs2243668 | | 0,997 | 0,980 | 1,015 | 7,65E-01 | G | NRP1 | 10 | 29071344 | www.ncbi.nlm.nih.gov/pubmed/29071344 |
| rs111629393 | | 0,993 | 0,981 | 1,006 | 2,85E-01 | G | NELL1 | 11 | 31969693 | www.ncbi.nlm.nih.gov/pubmed/31969693 |
| rs58621819 | | 0,966 | 0,953 | 0,980 | **1,57E-06** | T | LTBP3 | 11 | 30718901 | www.ncbi.nlm.nih.gov/pubmed/30718901 |
| rs1504722 | | 0,987 | 0,975 | 0,999 | 2,98E-02 | G | CNTN5 | 11 | 31969693 | www.ncbi.nlm.nih.gov/pubmed/31969693 |
| rs2458167 | | 1,013 | 1,000 | 1,025 | 4,19E-02 | A | CNTN5 | 11 | 30643256 | www.ncbi.nlm.nih.gov/pubmed/30643256 |
| rs586533 | | 1,013 | 1,001 | 1,026 | 3,78E-02 | G | CNTN5 | 11 | 29942085 | www.ncbi.nlm.nih.gov/pubmed/29942085 |
| rs1940709 | | 0,985 | 0,972 | 0,998 | 2,59E-02 | A | NCAM1 | 11 | 29942085 | www.ncbi.nlm.nih.gov/pubmed/29942085 |
| rs1940728 | | 0,978 | 0,967 | 0,990 | **2,18E-04** |  | NCAM1 | 11 | 29942085 | www.ncbi.nlm.nih.gov/pubmed/29942085 |
| rs72995548 | | 1,034 | 1,010 | 1,059 | 4,92E-03 |  | NCAM1, NCAM1-AS1 | 11 | 29942085 | www.ncbi.nlm.nih.gov/pubmed/29942085 |
| rs7174755 | | 0,996 | 0,984 | 1,008 | 5,24E-01 | T | ITGA11 | 15 | 23377640 | www.ncbi.nlm.nih.gov/pubmed/23377640 |
| rs3825992 | | 1,003 | 0,971 | 1,036 | 8,46E-01 | T | MFGE8 | 15 | 34099189 | www.ncbi.nlm.nih.gov/pubmed/34099189 |
| rs1560312 | | 1,006 | 0,994 | 1,018 | 3,66E-01 | G | MAPT | 17 | 29942085 | www.ncbi.nlm.nih.gov/pubmed/29942085 |
| rs9904290 | | 1,010 | 0,998 | 1,022 | 1,19E-01 | A | MAPT | 17 | 29942085 | www.ncbi.nlm.nih.gov/pubmed/29942085 |
| rs754593 | | 1,004 | 0,993 | 1,016 | 4,86E-01 | G | MAPT | 17 | 29942085 | www.ncbi.nlm.nih.gov/pubmed/29942085 |
| rs2435200 | | 0,986 | 0,975 | 0,998 | 1,87E-02 | A | MAPT | 17 | 29942085 | www.ncbi.nlm.nih.gov/pubmed/29942085 |
| rs8067056 | | 1,013 | 1,001 | 1,025 | 2,95E-02 | C | MAPT | 17 | 29942085 | www.ncbi.nlm.nih.gov/pubmed/29942085 |

TABLE S3: Binary regression analysis of the association between SNPs and co-morbid traits.

Analysis was adjusted for age, sex and first ten genetic principal components. P-values marked with bold indicate statistically significant p-values (p-value<0,05/38=0,001).

| Co-MORBID TRAITS | | | | | | |
| --- | --- | --- | --- | --- | --- | --- |
| SNP | Odds ratio | Lower CI | Upper CI | P-value | Associated genes | CHR |
| rs10746509 | 1,027 | 0,928 | 1,136 | 0,608 | GALNT2 | 1 |
| rs2042772 | 1,036 | 0,924 | 1,162 | 0,542 | LY75-CD302, LY75 | 2 |
| rs7374731 | 1,051 | 0,940 | 1,175 | 0,383 | DAG1 | 3 |
| rs4625 | 1,098 | 0,984 | 1,224 | 0,094 | DAG1 | 3 |
| rs9825823 | 1,042 | 0,944 | 1,151 | 0,413 | FHIT | 3 |
| rs141954845 | 1,031 | 0,932 | 1,141 | 0,550 | FHIT | 3 |
| rs7432943 | 0,974 | 0,880 | 1,077 | 0,603 | FHIT | 3 |
| rs935526 | 1,005 | 0,906 | 1,116 | 0,921 | ROBO2 | 3 |
| rs775766 | 0,974 | 0,874 | 1,086 | 0,633 | ROBO2 | 3 |
| rs114216628 | 1,022 | 0,633 | 1,651 | 0,928 | ROBO1 | 3 |
| rs17382228 | 1,016 | 0,911 | 1,133 | 0,772 | SORCS2 | 4 |
| rs6822806 | 1,054 | 0,909 | 1,223 | 0,485 | UNC5C | 4 |
| rs74378198 | 1,019 | 0,780 | 1,331 | 0,890 | OSMR | 5 |
| rs10514108 | 1,045 | 0,881 | 1,240 | 0,611 | ARSB | 5 |
| rs58825580 | 1,021 | 0,887 | 1,175 | 0,776 | BTN3A2 | 6 |
| rs13218591 | 0,967 | 0,868 | 1,077 | 0,542 | BTN3A2 | 6 |
| rs41316748 | 1,077 | 0,830 | 1,398 | 0,576 | TNXB | 6 |
| rs1150757 | 1,044 | 0,900 | 1,210 | 0,571 | TNXB | 6 |
| rs2269426 | 1,032 | 0,931 | 1,145 | 0,549 | TNXB | 6 |
| rs116078874 | 0,918 | 0,650 | 1,297 | 0,626 | GLO1 | 6 |
| rs3823624 | 0,979 | 0,860 | 1,114 | 0,745 | MAD1L1 | 7 |
| rs144631932 | 0,671 | 0,415 | 1,084 | 0,103 | ENPP2 | 8 |
| rs2243668 | 0,847 | 0,720 | 0,997 | 0,046 | NRP1 | 10 |
| rs111629393 | 1,097 | 0,984 | 1,222 | 0,095 | NELL1 | 11 |
| rs58621819 | 0,970 | 0,861 | 1,093 | 0,621 | LTBP3 | 11 |
| rs1504722 | 0,982 | 0,887 | 1,087 | 0,722 | CNTN5 | 11 |
| rs2458167 | 0,985 | 0,886 | 1,096 | 0,787 | CNTN5 | 11 |
| rs586533 | 0,983 | 0,883 | 1,093 | 0,747 | CNTN5 | 11 |
| rs1940709 | 1,019 | 0,909 | 1,143 | 0,747 | NCAM1 | 11 |
| rs1940728 | 1,018 | 0,920 | 1,127 | 0,729 | NCAM1 | 11 |
| rs72995548 | 0,953 | 0,784 | 1,158 | 0,625 | NCAM1, NCAM1-AS1 | 11 |
| rs7174755 | 1,059 | 0,955 | 1,173 | 0,277 | ITGA11 | 15 |
| rs3825992 | 1,195 | 0,925 | 1,545 | 0,173 | MFGE8 | 15 |
| rs1560312 | 0,948 | 0,854 | 1,053 | 0,320 | MAPT | 17 |
| rs9904290 | 1,001 | 0,904 | 1,108 | 0,989 | MAPT | 17 |
| rs754593 | 0,955 | 0,864 | 1,056 | 0,372 | MAPT | 17 |
| rs2435200 | 1,107 | 1,000 | 1,225 | 0,049 | MAPT | 17 |
| rs8067056 | 0,946 | 0,854 | 1,048 | 0,290 | MAPT | 17 |

TABLE S4: Binary regression analysis of the association between genetic risk score for depression (GRSdep) and depression phenotype.

Analysis was adjusted for age, sex and first ten genetic principal components. Number of significant SNPs=8, number of all SNPs=38. P-values marked with bold indicate statistically significant p-values (p-value<0,05/38=0,001).

| DEPRESSION | | | | | |  |
| --- | --- | --- | --- | --- | --- | --- |
| Outcome variable | Variable | Odds ratio | Lower CI | Upper CI | P-value | P-interaction (GRSdep x sex) |
| Depression | GRSdep-sig | 1,020 | 1,016 | 1,023 | **1,42E-24** | 0,172 |
|  | GRSdep-all | 1,009 | 1,007 | 1,011 | **7,47E-24** | 0,024 |

TABLE S5: Binary regression analysis of the association between genetic risk score for depression (GRSdep) and co-morbid traits.

Analysis was adjusted for age, sex and first ten genetic principal components. Number of significant SNPs=8, number of all SNPs=38.

| Co-MORBID TRAITS | | | | | |  |
| --- | --- | --- | --- | --- | --- | --- |
| Outcome variable | Variable | Odds ratio | Lower CI | Upper CI | P-value | P-interaction (GRSdep x sex) |
| Co-morbid traits | GRSdep-sig | 1,003 | 0,972 | 1,036 | 0,844 | 0,611 |
|  | GRSdep-all | 0,995 | 0,980 | 1,010 | 0,477 | 0,834 |

TABLE S6: Binary regression analysis of the association between quartiles of genetic risk score for depression (GRSdep) and depression phenotype.

Analysis was adjusted for age, sex and first ten genetic principal components. Number of significant SNPs=8, number of all SNPs=38. P-values marked with bold indicate statistically significant p-values (p-value<0,05/38=0,001).

|  | DEPRESSION | | | | | | |
| --- | --- | --- | --- | --- | --- | --- | --- |
| Outcome variable | | Variable | Odds ratio | Lower CI | Upper CI | P-value | P-interaction (GRSdep x sex) |
| Depression | | Quartile of GRSdep-sig |  |  |  | **1,27E-20** | 0,606 |
|  |  | Quartile of GRSdep-sig (1) | 1,041 | 1,018 | 1,064 | **4,91E-04** | 0,432 |
|  |  | Quartile of GRSdep-sig (2) | 1,070 | 1,043 | 1,099 | **3,97E-07** | 0,348 |
|  |  | Quartile of GRSdep-sig (3) | 1,118 | 1,092 | 1,144 | **6,86E-21** | 0,189 |
|  |  | Quartile of GRSdep-all |  |  |  | **9,21E-18** | 0,009 |
|  |  | Quartile of GRSdep-all (1) | 1,040 | 1,016 | 1,065 | 1,13E-03 | 0,596 |
|  |  | Quartile of GRSdep-all (2) | 1,054 | 1,030 | 1,079 | **1,06E-05** | 0,306 |
|  |  | Quartile of GRSdep-all (3) | 1,108 | 1,083 | 1,133 | **4,27E-19** | 0,010 |

TABLE S7: Sex-stratified binary regression analysis of the association between genetic risk score for depression (GRSdep) and depression phenotype.

Analysis was adjusted for age, sex and first ten genetic principal components. Number of significant SNPs=8, number of all SNPs=38. P-values marked with bold indicate statistically significant p-values (p-value<0,05/38=0,001).

| Sex-stratified analysis for depression | | | | | | | |
| --- | --- | --- | --- | --- | --- | --- | --- |
| Outcome variable | Variable | Sex | Odds ratio | Lower CI | Upper CI | P-value |  |
| Depression | GRSdep-sig | Men | 1,023 | 1,017 | 1,029 | **1,41E-14** |  |
|  |  | Women | 1,017 | 1,012 | 1,022 | **5,70E-12** |  |
|  | GRSdep-all | Men | 1,011 | 1,009 | 1,014 | **1,26E-16** |  |
|  |  | Women | 1,007 | 1,005 | 1,009 | **6,09E-10** |  |

TABLE S8: Sex-stratified binary regression analysis of the association between genetic risk score for depression (GRSdep) and co-morbid traits.

Analysis was adjusted for age, sex and first ten genetic principal components. Number of significant SNPs=8, number of all SNPs=38.

| Sex-stratified analysis for co-morbid traits | | | | | | |
| --- | --- | --- | --- | --- | --- | --- |
| Outcome variable | Variable | Sex | Odds ratio | Lower CI | Upper CI | P-value |
| Co-morbid traits | GRSdep-sig | Men | 0,988 | 0,943 | 1,034 | 5,93E-01 |
|  |  | Women | 1,019 | 0,974 | 1,065 | 4,18E-01 |
|  | GRSdep-all | Men | 0,996 | 0,975 | 1,017 | 7,14E-01 |
|  |  | Women | 0,994 | 0,973 | 1,015 | 5,49E-01 |

TABLE S9: Binary regression analysis of the association between quartiles of genetic risk score for depression (GRSdep) and co-morbid traits.

Analysis was adjusted for age, sex and first ten genetic principal components. Number of significant SNPs=8, number of all SNPs=38.

| Co-MORBID TRAITS | | | | | |  |
| --- | --- | --- | --- | --- | --- | --- |
| Outcome variable | Variable | Odds ratio | Lower CI | Upper CI | P-value | P-interaction (GRSdep x sex) |
| Co-morbid | Quartile of GRSdep-sig |  |  |  | 0,631 | 0,902 |
|  | Quartile of GRSdep-sig (1) | 1,087 | 0,899 | 1,313 | 0,391 | 0,555 |
|  | Quartile of GRSdep-sig (2) | 0,988 | 0,786 | 1,241 | 0,917 | 0,876 |
|  | Quartile of GRSdep-sig (3) | 0,976 | 0,797 | 1,195 | 0,813 | 0,513 |
|  | Quartile of GRSdep-all |  |  |  | 0,741 | 0,384 |
|  | Quartile of GRSdep-all (1) | 1,008 | 0,826 | 1,230 | 0,935 | 0,100 |
|  | Quartile of GRSdep-all (2) | 1,016 | 0,833 | 1,239 | 0,875 | 0,348 |
|  | Quartile of GRSdep-all (3) | 0,922 | 0,758 | 1,120 | 0,413 | 0,683 |

TABLE S10: Sex-stratified binary regression analysis of the association between quartiles of genetic risk score for depression (GRSdep) and co-morbid traits.

Analysis was adjusted for age, sex and first ten genetic principal components. Number of only significant SNPs=8, number of all SNPs=38.

| Sex-stratified analysis for co-morbid traits | | | | | | | |
| --- | --- | --- | --- | --- | --- | --- | --- |
| Outcome variable | Sex | Variable | Odds ratio | Lower CI | Upper CI | P-value |  |
| Co-morbid | males | Quartile of GRSdep-sig |  |  |  | 0,830 |  |
|  |  | Quartile of GRSdep-sig (1) | 1,024 | 0,783 | 1,340 | 0,862 |  |
|  |  | Quartile of GRSdep-sig (2) | 0,967 | 0,701 | 1,334 | 0,839 |  |
|  |  | Quartile of GRSdep-sig (3) | 0,908 | 0,680 | 1,212 | 0,511 |  |
|  | females | Quartile of GRSdep-sig |  |  |  | 0,689 |  |
|  |  | Quartile of GRSdep-sig (1) | 1,153 | 0,883 | 1,507 | 0,296 |  |
|  |  | Quartile of GRSdep-sig (2) | 1,011 | 0,731 | 1,397 | 0,949 |  |
|  |  | Quartile of GRSdep-sig (3) | 1,048 | 0,788 | 1,394 | 0,747 |  |
|  | males | Quartile of GRSdep-all |  |  |  | 0,398 |  |
|  |  | Quartile of GRSdep-all (1) | 1,193 | 0,898 | 1,585 | 0,224 |  |
|  |  | Quartile of GRSdep-all (2) | 1,121 | 0,841 | 1,495 | 0,436 |  |
|  |  | Quartile of GRSdep-all (3) | 0,960 | 0,720 | 1,281 | 0,782 |  |
|  | females | Quartile of GRSdep-all |  |  |  | 0,727 |  |
|  |  | Quartile of GRSdep-all (1) | 0,857 | 0,647 | 1,134 | 0,280 |  |
|  |  | Quartile of GRSdep-all (2) | 0,931 | 0,708 | 1,225 | 0,611 |  |
|  |  | Quartile of GRSdep-all (3) | 0,894 | 0,685 | 1,166 | 0,407 |  |

TABLE S11: Sex-stratified binary regression analysis of the association between quartiles of genetic risk score for depression (GRSdep) and depression phenotype.

Analysis was adjusted for age, sex and first ten genetic principal components. Number of significant SNPs=8, number of all SNPs=38. P-values marked with bold indicate statistically significant p-values (p-value<0,05/38=0,001).

| Sex-stratified analysis for depression | | | | | | | |
| --- | --- | --- | --- | --- | --- | --- | --- |
| Outcome variable | Sex | Variable | Odds ratio | Lower CI | Upper CI | P-value |  |
| Depression | males | Quartile of GRSdep-sig |  |  |  | **1,02E-11** |  |
|  |  | Quartile of GRSdep-sig (1) | 1,052 | 1,016 | 1,090 | 4,10E-03 |  |
|  |  | Quartile of GRSdep-sig (2) | 1,087 | 1,044 | 1,132 | **5,49E-05** |  |
|  |  | Quartile of GRSdep-sig (3) | 1,139 | 1,099 | 1,181 | **1,30E-12** |  |
|  | females | Quartile of GRSdep-sig |  |  |  | **1,88E-09** |  |
|  |  | Quartile of GRSdep-sig (1) | 1,033 | 1,003 | 1,063 | 3,19E-02 |  |
|  |  | Quartile of GRSdep-sig (2) | 1,059 | 1,023 | 1,096 | 1,21E-03 |  |
|  |  | Quartile of GRSdep-sig (3) | 1,103 | 1,070 | 1,137 | **3,37E-10** |  |
|  | males | Quartile of GRSdep (all SNPs) |  |  |  | **1,10E-14** |  |
|  |  | Quartile of GRSdep-all (1) | 1,032 | 0,995 | 1,071 | 8,83E-02 |  |
|  |  | Quartile of GRSdep-all (2) | 1,070 | 1,032 | 1,110 | **2,67E-04** |  |
|  |  | Quartile of GRSdep-all (3) | 1,148 | 1,108 | 1,188 | **8,44E-15** |  |
|  | females | Quartile of GRSdep-all |  |  |  | **8,29E-06** |  |
|  |  | Quartile of GRSdep-all (1) | 1,046 | 1,014 | 1,079 | 4,54E-03 |  |
|  |  | Quartile of GRSdep-all (2) | 1,043 | 1,012 | 1,076 | 6,99E-03 |  |
|  |  | Quartile of GRSdep-all (3) | 1,080 | 1,049 | 1,112 | **3,01E-07** |  |

# Section S1: Definition of depression cases and controls

We identified five measures for depression. Participants who met the criteria for at least two of the five depression measures were defined as depression cases. The five depression measures are described below.

**Help-seeking cases:**

UK Biobank participants reported information at baseline or the subsequent two repeat assessments. Participants were classified as ‘help-seeking’ cases if they endorsed either of the following questions:

- “Have you ever seen a general practitioner for nerves, anxiety, tension or depression?” [Field ID 2090]

#### “Have you ever seen a psychiatrist for nerves, anxiety, tension or depression?” [Field ID 2100]

#### Depression cases according to the definition by Smith et al ^1^

#### UK Biobank participants reported information at baseline or the subsequent two repeat assessments. Participants were classified as depression (Smith) cases if they endorsed ‘help-seeking’ and also met the additional criteria defined by Smith et al.

#### “Have you ever seen a general practitioner for nerves, anxiety, tension or depression?” [Field ID 2090] OR “Have you ever seen a psychiatrist for nerves, anxiety, tension or depression?” [Field ID 2100] AND

- “Looking back over your life, have you ever had a time when you were feeling depressed or down for at least a whole week?” (Field ID: 4598)
- “How many weeks was the longest period when you were feeling depressed or down” (Field ID: 4609) - at least 2 weeks duration

**Hospital (ICD-10) cases:**

#### Participants were classified as ‘hospital (ICD-10)’ cases if they were assigned a primary [Field ID 41202] or secondary [Field ID 41204] ICD-10 code for depressive episode (F32–F32,9) or recurrent depressive disorder (F33–F33,9) from linked hospital admission records between April 1997 and October 2016:

#### Depressive episode: F32, F320, F321, F322, F323, F328, F329.

#### Recurrent depressive disorder: F33, F330, F331, F332, F333, F334, F338, F339.

#### Self-reported depression cases:

Participants were classified as ‘self-reported depression’ cases if they endorsed having experienced depression (past or present) at baseline or the subsequent two repeat assessments [Field ID 20002, UK Biobank (UKB) Data-Coding 6 = 1286].

#### Antidepressant usage cases:

Participants were classified as ‘antidepressant usage’ cases if they endorsed currently taking antidepressant medications at baseline or the subsequent two repeat assessments [ID 20003, UKB Data-Coding 4]:

- Antidepressant codes: 1140879616, 1140921600, 1140879540, 1140867878, 1140916282, 1140909806, 1140867888, 1141152732, 1141180212, 1140879634, 1140867876, 1140882236, 1141190158, 1141200564, 1140867726, 1140879620, 1140867818, 1140879630, 1140879628, 1141151946, 1140867948, 1140867624, 1140867756, 1140867884, 1141151978, 1141152736, 1141201834, 1140867690, 1140867640, 1140867920, 1140867850, 1140879544, 1141200570, 1140867934, 1140867758, 1140867914, 1140867820, 1141151982, 1140882244, 1140879556, 1140867852, 1140867860, 1140917460, 1140867938, 1140867856, 1140867922, 1140910820, 1140882312, 1140867944, 1140867784, 1140867812, 1140867668

**Controls:**

The participants who did not meet the criteria for any of the above mentioned five depression measures were classified as controls. Moreover, the participants who did not endorse the following question for depression in the Mental Health Questionnaire were also classified as controls:

- “Have you been diagnosed with one or more of the following mental health problems by a professional, even if you don’t have it currently?” [ID 20544, UKB Data-Coding 1401 = 11]

# Section S2: Definition of co-morbid cases and controls

Both self-reported data and ICD 10 codes were used to identify cases and controls.

**Cases:**

Participants were classified as cases if they if they were assigned a primary [Field ID 41202] or secondary [Field ID 41204] ICD-10 code from linked hospital admission records between April 1997 and October 2016

for:

- Anxiety (F40-F41),
- Bipolar disorder (F30-F31)
- Schizoaffective disorder (F30-F31)

**AND/OR**

If they had a self-reported non-cancer illness code [Field ID 20002] for:

- Anxiety (code:1287)
- Bipolar disorder (code:1291)
- Schizophrenia (code:1289)

**Controls**

Controls for all three co-morbid traits were participants who did not meet any criteria for anxiety, bipolar disorder or schizophrenia.^1^

# Section S3: Transcriptome analyses

Transcriptome analysis was conducted for identified depression-related genes based on the results from the UK biobank data. For this analysis, we used three publicly-available cohorts (GSE53987, GSE98793, and GSE46743) deposited in the Gene Expression Omnibus (GEO) ^2^. Cohort GSE53987 comprises brain sample data for major depressive disorder (MDD), schizophrenia, bipolar disorder, and controls. Sample collection and preparation are provided in the original publication ^3^. For each disease status, data from three brain tissues was available and included the associative striatum, hippocampus, and prefrontal cortex (Brodmann area 46). We compared transcriptome profiles of MDD patients (n = 16 – 17) versus controls (n = 18 – 19) separately for each of the three brain tissues. The second cohort (GSE98793) includes data from the whole blood samples of 128 MDD patients (64 were comorbid for generalized anxiety disorder) and 64 healthy controls ^4^. The last cohort represents a fraction of data from GSE46743. The initial study objective was to investigate the differences in blood gene expression in depressed (n=69) individuals versus controls (n=91) in the context of glucocorticoid receptor (GR) activation. The study has been performed on male subjects. For further information, please refer to the initial article ^5^. Expression data was available before and after stimulation with a GR-stimulating agent (dexamethasone). In the context of this work, we only used the data obtained prior to stimulation (baseline).

All data preparation and analysis steps were performed in R environment (version 4.2.0). Cohorts GSE53987 and GSE98793 include data derived from Affymetrix Human Genome U133 Plus 2,0 Array, whereas GSE46743 is based on Illumina HumanHT-12 V3,0 expression beadchip, and thus preprocessing steps were different depending on the input data. For the first two cohorts, data files were available from the raw CEL format. Reading, quality control, preprocessing, and data normalization for these cohorts were performed using the *affy*-based framework for Affymetrix arrays ^6^. Briefly, data was imported with the *ReadAffy*function, and chip images were previewed with the *image* method implemented within the package to detect potential artifacts. Then, all preprocessing and normalization steps were performed within the *expresso* pipeline. Signal intensities were corrected using a robust multiarray average (RMA) method ^7^, and then log2-transformed values were quantile normalized. A probe-wise correction was based only on *perfect match*^8^ intensities (*pmonly*). The resulting summary expression values were obtained using the *medianpolish*procedure ^9^. The aforementioned steps were performed independently for every brain tissue for the cohort GSE53987. The data for GSE46743 was obtained directly from GEO. We used adjusted data, where expression signals were adjusted through variance stabilization and normalization (VSN), and batches were corrected with *ComBat* with fixed effects of amplification round ^10^ as clarified in the initial publication ^5^.

Differential expression (DE) analysis was conducted only for probes located in the targeted genes with significant depression-related SNPs that passed quality control steps and were available for analysis. The initial list included six genes: Dystroglycan 1 (DAG1), Fragile Histidine Triad Diadenosine Triphosphatase (FHIT), Butyrophilin Subfamily 3 Member A2 (BTN3A2), Tenascin XB (TNXB), Latent Transforming Growth Factor Beta Binding Protein 3 (LTBP3), and Neural Cell Adhesion Molecule 1 (NCAM1). Identifying relevant transcripts was performed using corresponding annotation files for arrays used in the analysis. For Affymetrix-based cohorts, all investigated genes had associated transcripts, whereas, for the Illumina-based array (GSE46743), no transcripts were available for TNXB. We used a *limma*-based analysis framework to identify DE probes in all three cohorts. “*Limma”* is an R package that uses linear models to identify DE probes, and the calculated T-statistics are moderated with the empirical Bayesian method ^11,12^. In the models, expression values were considered as a quantitative dependent variable, while the diagnosis or disease status was used as a binary predictor (Yes/No). All linear models were adjusted for confounders that were selected depending on a particular cohort and available phenotypical data. In GSE53987, gender (Male/Female), body mass index (BMI, numeric), age (numeric), race (Black/White), tissue pH (numeric), post-mortem interval (numeric), and RNA integrity number (numeric) were used as confounders. Analysis in GSE98793 was corrected for gender (Male/Female), age (numeric), and anxiety status (Yes/No). In the last cohort, we adjusted models only for BMI (numeric) and age (numeric) since all participants were male. In all analyses, we considered nominally significant results, however, false-discovery-rate-adjusted p-values and family-wise error rates arealso reported. We did not set any threshold for log2 fold change.

# Section S4: DNA methylation analyses

Methylation analysis was conducted to identify potential changes in methylation among depression-related genes. For this purpose, we used data from three independent cohorts. The first cohort (PSY-SCR) includes 221 non-related adolescents aged 14-16 that were recruited from public schools in Uppsala County, Sweden between 2012 and 2013. This study aims to identify genetic and epigenetic markers in whole blood related to the risk of developing a psychiatric disease in healthy adolescents. Upon arrival, participants passed through the series of questionaries and reported their height, age, and gender. The body weight of participants was measured to calculate BMI, and a whole blood sample was collected for DNA extraction and methylation analysis. For further details regarding sample procedures, please refer to our previous publications ^13,14^. The computer-based Development and Well-Being Assessment (DAWBA) questionnaire was used to determine the risk to develop depression in the studied individuals. The risk for depression was categorized in six probability band scores, i.e. 0 (<0,1%), 1 (~0,5%), 2 (~3%), 3 (~15%), 4 (~50%), and 5 (>70%) ^15^. We defined a “low-risk” group as individuals with a risk of less than 50%, whereas the “high-risk” group included participants with a depression risk ≥ 50%. Thus, the resulting cohort included 24 individuals with high depression risk and 197 individuals with low depression risk. The other two cohorts are publicly available and could be found on GEO (GSE72680 and GSE125105). The cohort GSE72680 represents a part of the Grady Trauma Project that was conducted in Atlanta, U.S. This study analyzed DNA methylation in whole blood in relation to psychiatric disease. Sample collection and initial phenotypical characterization are described in detail in the initial publication ^16^. Initial phenotypical data includes multiple psychiatric risk scores, such as Beck Depression Inventory (BDI) ^17^, Childhood Trauma Questionnaire ^18^, and several life stress scores. Additionally, information regarding treatment for depression, anxiety, bipolar disorder, or post-traumatic stress disorder is also available for most of the individuals. Individuals with depression were identified by BDI total score (>=21). The initial cohort includes 422 participants, of which 312 were amenable for analysis (100 depressed and 212 non-depressed) after the removal of participants with missing phenotypical data. The last cohort (GSE125105) was recruited at the Max Planck Institute of Psychiatry and includes the whole blood methylation data from 489 depressed and 210 control subjects. Sample collection and phenotypical characterization information are provided elsewhere ^19,20^. After removing samples with missing data, 324 depressed and 167 non-depressed participants were included in the analysis.

Methylation data for all three cohorts was obtained using Illumina HumanMethylation450 array that captures approximately 450 000 different CpG sites. All data preparation and analysis steps were performed in the R environment (version 4.2.0). DNA methylation data for PSY-SCR and GSE125105 was available in the form of raw IDAT files. We used a *minfi*-based framework ^21^ for data handling. Background correction was performed, using a “noob” method ^22^. Beta values were quantile-normalized and subsequently corrected for type I and type II probe bias via Beta Mixture Quantile Dilation (BMIQ) ^23^ from the *wateRmelon* package ^24^. We used the “ComBat” function from the “*sva*” package to adjust for technical variations ^25,26^. Additionally, we used a *minfi*-based implementation of the Houseman algorithm to correct the methylation data for white peripheral blood cell heterogeneity (CD4+, CD8+, natural killer cells, b-cells, monocytes, and granulocytes) ^27^, applying a linear model-based approach as was described previously ^28^. Methylation data for GSE72680 was processed starting from raw beta values deposited in GEO. We performed a quantile normalization of these values followed by BMIQ as was done for two other cohorts, and the batch effect was corrected with the *“ComBat”.* Cell proportions were available from phenotypical data in GEO. Before identification of gene-related probes and analysis, we performed several filtering steps on available CpG list to remove non-reliable probes. We kept the samples where more than 75% of samples have a detection p-value less than 0,00005 and probes where more than 75% of samples have a detection p-value less than 0,01. Probes from sex chromosomes, non-CpGs, and probes with missing beta values were excluded. We removed all probes that have an SNP with a minor allele frequency (MAF) higher than 5% within the probe sequence. All probes that have an SNP at the CpG site and a single-base extension were excluded. Additionally, we removed cross-reactive probes identified by Chen et al. ^29^ and Benton et al ^30^.

Methylation analysis was performed only for probes associated with six investigated genes. Identification of relevant CpG sites was done, utilizing Illumina annotation files for the HumanMethylation450 array. We updated gene names listed in the array, using the last available version (June 2022) of the gene synonyms and names dataset from the National Center for Biotechnology Information's (NCBI) Gene database ^31^. Methylation analysis for promoter-associated CpGs and gene body CpGs was done separately. Identification of promoter-related CpGs was achieved with Illumina annotation files, using keywords “5’UTR” and “TSS”. Gene body CpGs were selected with the keyword “Body” instead. In total, 102 CpGs for GSE72680 and 137 CpGs for PSY-SCR and GSE125105 were included in the promoter analysis, whereas 300 CpGs for GSE72680 and 459 CpGs for PSY-SCR and GSE125105 were classified as gene body CpGs of investigated genes. Similar to transcriptome analysis, we used the *“Limma”* package to identify differentially methylated sites. In the models, methylation values (M-values ^32^) were considered as a quantitative dependent variable, while the diagnosis or disease status was used as a binary predictor (Yes/No). Models were adjusted for confounders that were selected depending on a particular cohort and the availability of phenotypical data. In the PSY-SCR cohort, we adjusted the models for gender (Male/Female), body mass index (BMI, numeric), age (numeric), and the time batch covariable (binary factor) since methylation was performed at two time points. In the cohort GSE72680, the model was corrected for treatment for depression (Yes/No), treatment for bipolar disorder (Yes/No), treatment for anxiety (Yes/No), gender (Male/Female), body mass index (BMI, numeric), age (numeric), ethnic background (factor with several levels), and cell proportions (numeric). Lastly, the model for GSE125105 was adjusted for gender (Male/Female), body mass index (BMI, numeric), age (numeric), and two principal components of the genotypes to account for an ethnic background as described previously ^16^. We considered nominally significant results that overlapped between cohorts, however, false-discovery-rate-adjusted p-values and family-wise error rates are also reported. Graph visualization of differentially methylated CpG sites, associated genes, and studied cohorts was done in R, using the *“visNetwork”* package.

# Section S5: Transcriptomic cohorts

In the tables below are characteristics of the cohorts used for transcriptome analysis. The cohort GSE53987 is split into three sub-tables, showing demographics and other data for each of the investigated brain tissues. Categorical variables are shown as counts (frequencies). Percentages correspond to the percent of participants within a sub-group. Numerical data is shown as mean ± standard deviation. Abbreviations: MDD, major depressive disorder; BA46, Brodmann area 46; BMI, body mass index.

**GSE53987**

| **GSE53987: Hippocampus** | | |
| --- | --- | --- |
| Initial datasetincludes 35 participants | | |
| Participants with missing data excluded: 0 Resulting number of participants: 35 | | |
| **Diagnosis** | **MDD: 17** | **Healthy: 18** |
| Gender | Female: 8 (47,1%) Male: 9 (52,9%) | Female: 9 (50%) Male: 9 (50%) |
| Ethnicalbackground | White: 16 (94,1%) Black: 1 (5,9%) | White: 17 (94,4%) Black: 1 (5,6%) |
| Age | 45,18 ± 10,71 Min: 26; Max: 62 | 48,17 ± 10,95 Min: 22; Max: 68 |
| **GSE53987: Pre-frontal cortex (BA46)** | | |
| Initial datasetincludes 36 participants | | |
| Participants with missing data excluded: 0 Resulting number of participants: 36 | | |
| **Diagnosis** | **MDD: 17** | **Healthy: 19** |
| Gender | Female: 8 (47,1%) Male: 9 (52,9%) | Female: 9 (47,4%) Male: 10 (52,6%) |
| Ethnicalbackground | White: 16 (94,1%) Black: 1 (5,9%) | White: 18 (94,7%) Black: 1 (5,3%) |
| Age | 45,18 ± 10,71 Min: 26; Max: 62 | 48,05 ± 10,65 Min: 22; Max: 68 |
| **GSE53987: Associative striatum** | | |
| Initial datasetincludes 34 participants | | |
| Participants with missing data excluded: 0 Resulting number of participants: 34 | | |
| **Diagnosis** | **MDD: 16** | **Healthy: 18** |
| Gender | Female: 6 (37,5%) Male: 10 (62,5%) | Female: 8 (44,4%) Male: 10 (55,6%) |
| Ethnicalbackground | White: 16 (100%) Black: 0 (0%) | White: 17 (94,4%) Black: 1 (5,6%) |
| Age | 46,5 ± 9,96 Min: 26; Max: 62 | 48,44 ± 10,82 Min: 22; Max: 68 |

**GSE98793**

| **GSE98793** | | |
| --- | --- | --- |
| Initial datasetincludes 192 participants | | |
| Participants with missing data excluded: 0 Resulting number of participants: 192 | | |
| **Diagnosis** | **MDD: 128** | **Healthy: 64** |
| Gender | Female: 96 (75%) Male: 32 (25%) | Female: 48 (75%) Male: 16 (25%) |
| Age | 52,04 ± 11,51 Min: 31; Max: 72,8 | 52,03 ± 11,41 Min: 31,2; Max: 73,1 |
| Anxiety | Yes: 64 (50%) No: 64 (50%) | Yes: 0 (0%) No: 64 (100%) |

**GSE46743**

| **GSE46743** | | |
| --- | --- | --- |
| Initial dataset includes 160 male participants at baseline | | |
| Participants with missing data excluded: 0 Resulting number of participants: 160 | | |
| **Diagnosis** | **MDD: 69** | **Healthy: 91** |
| Age | 48,39 ± 13,59 Min: 21; Max: 73 | 40,18 ± 12,45 Min: 18; Max: 61 |
| BMI | 26,05 ± 3,61 Min: 19,88; Max: 40,15 | 24,93 ± 3,14 Min: 19,59; Max: 37,45 |

# Section S6: Methylation cohorts

In the tables below are characteristics of the cohorts used for differential methylation analysis. Categorical variables are shown as counts (frequencies). Percentages correspond to the percent of participants within a sub-group. Numerical data is shown as mean ± standard deviation. Abbreviations: DAWBA, Development and Well-Being Assessment questionnaire; BMI, body mass index; BDI, Beck Depression Inventory; BP, bipolar disorder; AD, anxiety disorder.

**PSY**

| **PSY** | | |
| --- | --- | --- |
| Initial datasetincludes 221 participants | | |
| Participants with missing data excluded: 0 | | |
| **Depression risk** | **Highdepr. risk: 24** | **Low depr. risk: 197** |
| DAWBA Depdand | 4,33 ± 0,48 Min: 4; Max: 5 | 0,9 ± 1,08 Min: 0; Max: 3 |
| Gender | Male: 2 (8,3%) Female: 22 (91,7%) | Male: 54 (27,4%) Female: 143 (72,6%) |
| Age | 15,42 ± 0,65 Min: 14; Max: 16 | 15,45 ± 0,63 Min: 14; Max: 17 |
| BMI | 22,51 ± 3,79 Min: 16,75; Max: 31,91 | 21,85 ± 3,39 Min: 15,65; Max: 37,54 |

**GSE72680**

| **GSE72680** | | |
| --- | --- | --- |
| Initial datasetincludes 422 participants | | |
| Participants with missing data excluded: 110 Resulting number of participants: 312 | | |
| **Depression category** | **Depressed: 100** | **Normal: 212** |
| BDI Total Score | 31,65 ± 9,1 Min: 21; Max: 58 | 8,93 ± 6,03 Min: 0; Max: 20 |
| Gender | Female: 70 (70%) Male: 30 (30%) | Female: 147 (69,3%) Male: 65 (30,7%) |
| Age | 41,67 ± 12,11 Min: 18; Max: 70 | 41,11 ± 13,22 Min: 18; Max: 74 |
| BMI | 32,62 ± 8,54 Min: 18,22; Max: 58,15 | 32,1 ± 8,24 Min: 18,93; Max: 72,97 |
| Ethnicalbackground | African American: 88 (88%) Caucasian: 8 (8%) Mixed: 3 (3%) Other: 1 (1%) | African American: 202 (95,3%) Caucasian: 10 (4,7%) Mixed: 0 (0%) Other: 0 (0%) |
| Depression treatment | Yes: 52 (52%) No: 48 (48%) | Yes: 58 (27,4%) No: 154 (72,6%) |
| BP treatment | Yes: 17 (17%) No: 83 (83%) | Yes: 15 (7,1%) No: 197 (92,9%) |
| AD treatment | Yes: 31 (31%) No: 69 (69%) | Yes: 21 (9,9%) No: 191 (90,1%) |

**GSE125105**

| **GSE125105** | | |
| --- | --- | --- |
| Initial datasetincludes 699 participants | | |
| Participants with missing data excluded: 208 Resulting number of participants: 491 | | |
| **Diagnosis** | **Depression: 324** | **Non-depressed: 167** |
| Age | 46,94 ± 13,53 Min: 17; Max: 79 | 49,42 ± 13,8 Min: 19; Max: 78 |
| Gender | Female: 183 (56,5%) Male: 141 (43,5%) | Female: 100 (59,9%) Male: 67 (40,1%) |
| BMI | 24,55 ± 4,12 Min: 15,03; Max: 45,86 | 24,17 ± 3,56 Min: 18,34; Max: 37,83 |

# Section S7: References

1. Smith, D. J. *et al.* Prevalence and characteristics of probable major depression and bipolar disorder within UK biobank: cross-sectional study of 172,751 participants. *PLoS One***8**, e75362 (2013).

2. Barrett, T. *et al.* NCBI GEO: archive for functional genomics data sets--update. *Nucleic Acids Res***41**, D991-995 (2013).

3. Lanz, T. A. *et al.* Postmortem transcriptional profiling reveals widespread increase in inflammation in schizophrenia: a comparison of prefrontal cortex, striatum, and hippocampus among matched tetrads of controls with subjects diagnosed with schizophrenia, bipolar or major depressive disorder. *Transl Psychiatry***9**, 151 (2019).

4. Leday, G. G. R. *et al.* Replicable and Coupled Changes in Innate and Adaptive Immune Gene Expression in Two Case-Control Studies of Blood Microarrays in Major Depressive Disorder. *Biol Psychiatry***83**, 70–80 (2018).

5. Arloth, J. *et al.* Genetic Differences in the Immediate Transcriptome Response to Stress Predict Risk-Related Brain Function and Psychiatric Disorders. *Neuron***86**, 1189–1202 (2015).

6. Gautier, L., Cope, L., Bolstad, B. M. & Irizarry, R. A. affy--analysis of Affymetrix GeneChip data at the probe level. *Bioinformatics***20**, 307–315 (2004).

7. Irizarry, R. A. *et al.* Exploration, normalization, and summaries of high density oligonucleotide array probe level data. *Biostatistics***4**, 249–264 (2003).

8. Binder, H. & Preibisch, S. GeneChip microarrays—signal intensities, RNA concentrations and probe sequences. *J. Phys.: Condens. Matter***18**, S537–S566 (2006).

9. Berger, F. & Carlon, E. From hybridization theory to microarray data analysis: performance evaluation. *BMC Bioinformatics***12**, 464 (2011).

10. Leek, J. T. & Storey, J. D. Capturing heterogeneity in gene expression studies by surrogate variable analysis. *PLoS Genet***3**, 1724–1735 (2007).

11. Ritchie, M. E. *et al.* limma powers differential expression analyses for RNA-sequencing and microarray studies. *Nucleic Acids Res***43**, e47 (2015).

12. Smyth, G. K. Linear models and empirical bayes methods for assessing differential expression in microarray experiments. *Stat Appl Genet Mol Biol***3**, Article3 (2004).

13. Ciuculete, D. M. *et al.* meQTL and ncRNA functional analyses of 102 GWAS-SNPs associated with depression implicate HACE1 and SHANK2 genes. *Clin Epigenetics***12**, 99 (2020).

14. Ciuculete, D. M. *et al.* Changes in methylation within the STK32B promoter are associated with an increased risk for generalized anxiety disorder in adolescents. *J Psychiatr Res***102**, 44–51 (2018).

15. Goodman, A., Heiervang, E., Collishaw, S. & Goodman, R. The ‘DAWBA bands’ as an ordered-categorical measure of child mental health: description and validation in British and Norwegian samples. *Soc Psychiatry Psychiatr Epidemiol***46**, 521–532 (2011).

16. Zannas, A. S. *et al.* Epigenetic upregulation of FKBP5 by aging and stress contributes to NF-κB-driven inflammation and cardiovascular risk. *Proc Natl Acad Sci U S A***116**, 11370–11379 (2019).

17. Beck, A. T., Steer, R. A. & Carbin, M. G. Psychometric properties of the Beck Depression Inventory: Twenty-five years of evaluation. *Clinical Psychology Review***8**, 77–100 (1988).

18. Bernstein, D. P. *et al.* Development and validation of a brief screening version of the Childhood Trauma Questionnaire. *Child Abuse Negl***27**, 169–190 (2003).

19. Arloth, J. *et al.* DeepWAS: Multivariate genotype-phenotype associations by directly integrating regulatory information using deep learning. *PLoS Comput Biol***16**, e1007616 (2020).

20. Moore, S. R. *et al.* Sex differences in the genetic regulation of the blood transcriptome response to glucocorticoid receptor activation. *Transl Psychiatry***11**, 632 (2021).

21. Aryee, M. J. *et al.* Minfi: a flexible and comprehensive Bioconductor package for the analysis of Infinium DNA methylation microarrays. *Bioinformatics***30**, 1363–1369 (2014).

22. Triche, T. J., Weisenberger, D. J., Van Den Berg, D., Laird, P. W. & Siegmund, K. D. Low-level processing of Illumina Infinium DNA Methylation BeadArrays. *Nucleic Acids Res***41**, e90 (2013).

23. Teschendorff, A. E. *et al.* A beta-mixture quantile normalization method for correcting probe design bias in Illumina Infinium 450 k DNA methylation data. *Bioinformatics***29**, 189–196 (2013).

24. Pidsley, R. *et al.* A data-driven approach to preprocessing Illumina 450K methylation array data. *BMC Genomics***14**, 293 (2013).

25. Leek, J. T., Johnson, W. E., Parker, H. S., Jaffe, A. E. & Storey, J. D. The sva package for removing batch effects and other unwanted variation in high-throughput experiments. *Bioinformatics***28**, 882–883 (2012).

26. Johnson, W. E., Li, C. & Rabinovic, A. Adjusting batch effects in microarray expression data using empirical Bayes methods. *Biostatistics***8**, 118–127 (2007).

27. Houseman, E. A. *et al.* DNA methylation arrays as surrogate measures of cell mixture distribution. *BMC Bioinformatics***13**, 86 (2012).

28. Jones, M. J., Islam, S. A., Edgar, R. D. & Kobor, M. S. Adjusting for Cell Type Composition in DNA Methylation Data Using a Regression-Based Approach. *Methods Mol Biol***1589**, 99–106 (2017).

29. Chen, Y. *et al.* Discovery of cross-reactive probes and polymorphic CpGs in the Illumina Infinium HumanMethylation450 microarray. *Epigenetics***8**, 203–209 (2013).

30. Benton, M. C. *et al.* An analysis of DNA methylation in human adipose tissue reveals differential modification of obesity genes before and after gastric bypass and weight loss. *Genome Biol***16**, 8 (2015).

31. Brown, G. R. *et al.* Gene: a gene-centered information resource at NCBI. *Nucleic Acids Res***43**, D36-42 (2015).

32. Du, P. *et al.* Comparison of Beta-value and M-value methods for quantifying methylation levels by microarray analysis. *BMC Bioinformatics***11**, 587 (2010).

1. Smith, D. J. *et al.* Prevalence and characteristics of probable major depression and bipolar disorder within UK biobank: cross-sectional study of 172,751 participants. *PLoS One***8**, e75362 (2013).

2. Barrett, T. *et al.* NCBI GEO: archive for functional genomics data sets--update. *Nucleic Acids Res***41**, D991-995 (2013).

3. Lanz, T. A. *et al.* Postmortem transcriptional profiling reveals widespread increase in inflammation in schizophrenia: a comparison of prefrontal cortex, striatum, and hippocampus among matched tetrads of controls with subjects diagnosed with schizophrenia, bipolar or major depressive disorder. *Transl Psychiatry***9**, 151 (2019).

4. Leday, G. G. R. *et al.* Replicable and Coupled Changes in Innate and Adaptive Immune Gene Expression in Two Case-Control Studies of Blood Microarrays in Major Depressive Disorder. *Biol Psychiatry***83**, 70–80 (2018).

5. Arloth, J. *et al.* Genetic Differences in the Immediate Transcriptome Response to Stress Predict Risk-Related Brain Function and Psychiatric Disorders. *Neuron***86**, 1189–1202 (2015).

6. Gautier, L., Cope, L., Bolstad, B. M. & Irizarry, R. A. affy--analysis of Affymetrix GeneChip data at the probe level. *Bioinformatics***20**, 307–315 (2004).

7. Irizarry, R. A. *et al.* Exploration, normalization, and summaries of high density oligonucleotide array probe level data. *Biostatistics***4**, 249–264 (2003).

8. Binder, H. & Preibisch, S. GeneChip microarrays—signal intensities, RNA concentrations and probe sequences. *J. Phys.: Condens. Matter***18**, S537–S566 (2006).

9. Berger, F. & Carlon, E. From hybridization theory to microarray data analysis: performance evaluation. *BMC Bioinformatics***12**, 464 (2011).

10. Leek, J. T. & Storey, J. D. Capturing heterogeneity in gene expression studies by surrogate variable analysis. *PLoS Genet***3**, 1724–1735 (2007).

11. Ritchie, M. E. *et al.* limma powers differential expression analyses for RNA-sequencing and microarray studies. *Nucleic Acids Res***43**, e47 (2015).

12. Smyth, G. K. Linear models and empirical bayes methods for assessing differential expression in microarray experiments. *Stat Appl Genet Mol Biol***3**, Article3 (2004).

13. Ciuculete, D. M. *et al.* meQTL and ncRNA functional analyses of 102 GWAS-SNPs associated with depression implicate HACE1 and SHANK2 genes. *Clin Epigenetics***12**, 99 (2020).

14. Ciuculete, D. M. *et al.* Changes in methylation within the STK32B promoter are associated with an increased risk for generalized anxiety disorder in adolescents. *J Psychiatr Res***102**, 44–51 (2018).

15. Goodman, A., Heiervang, E., Collishaw, S. & Goodman, R. The ‘DAWBA bands’ as an ordered-categorical measure of child mental health: description and validation in British and Norwegian samples. *Soc Psychiatry Psychiatr Epidemiol***46**, 521–532 (2011).

16. Zannas, A. S. *et al.* Epigenetic upregulation of FKBP5 by aging and stress contributes to NF-κB-driven inflammation and cardiovascular risk. *Proc Natl Acad Sci U S A***116**, 11370–11379 (2019).

17. Beck, A. T., Steer, R. A. & Carbin, M. G. Psychometric properties of the Beck Depression Inventory: Twenty-five years of evaluation. *Clinical Psychology Review***8**, 77–100 (1988).

18. Bernstein, D. P. *et al.* Development and validation of a brief screening version of the Childhood Trauma Questionnaire. *Child Abuse Negl***27**, 169–190 (2003).

19. Arloth, J. *et al.* DeepWAS: Multivariate genotype-phenotype associations by directly integrating regulatory information using deep learning. *PLoS Comput Biol***16**, e1007616 (2020).

20. Moore, S. R. *et al.* Sex differences in the genetic regulation of the blood transcriptome response to glucocorticoid receptor activation. *Transl Psychiatry***11**, 632 (2021).

21. Aryee, M. J. *et al.* Minfi: a flexible and comprehensive Bioconductor package for the analysis of Infinium DNA methylation microarrays. *Bioinformatics***30**, 1363–1369 (2014).

22. Triche, T. J., Weisenberger, D. J., Van Den Berg, D., Laird, P. W. & Siegmund, K. D. Low-level processing of Illumina Infinium DNA Methylation BeadArrays. *Nucleic Acids Res***41**, e90 (2013).

23. Teschendorff, A. E. *et al.* A beta-mixture quantile normalization method for correcting probe design bias in Illumina Infinium 450 k DNA methylation data. *Bioinformatics***29**, 189–196 (2013).

24. Pidsley, R. *et al.* A data-driven approach to preprocessing Illumina 450K methylation array data. *BMC Genomics***14**, 293 (2013).

25. Leek, J. T., Johnson, W. E., Parker, H. S., Jaffe, A. E. & Storey, J. D. The sva package for removing batch effects and other unwanted variation in high-throughput experiments. *Bioinformatics***28**, 882–883 (2012).

26. Johnson, W. E., Li, C. & Rabinovic, A. Adjusting batch effects in microarray expression data using empirical Bayes methods. *Biostatistics***8**, 118–127 (2007).

27. Houseman, E. A. *et al.* DNA methylation arrays as surrogate measures of cell mixture distribution. *BMC Bioinformatics***13**, 86 (2012).

28. Jones, M. J., Islam, S. A., Edgar, R. D. & Kobor, M. S. Adjusting for Cell Type Composition in DNA Methylation Data Using a Regression-Based Approach. *Methods Mol Biol***1589**, 99–106 (2017).

29. Chen, Y. *et al.* Discovery of cross-reactive probes and polymorphic CpGs in the Illumina Infinium HumanMethylation450 microarray. *Epigenetics***8**, 203–209 (2013).

30. Benton, M. C. *et al.* An analysis of DNA methylation in human adipose tissue reveals differential modification of obesity genes before and after gastric bypass and weight loss. *Genome Biol***16**, 8 (2015).

31. Brown, G. R. *et al.* Gene: a gene-centered information resource at NCBI. *Nucleic Acids Res***43**, D36-42 (2015).

32. Du, P. *et al.* Comparison of Beta-value and M-value methods for quantifying methylation levels by microarray analysis. *BMC Bioinformatics***11**, 587 (2010).
